# Parallel Computational Subunits in Dentate Granule Cells Generate Multiple Place Fields

**DOI:** 10.1371/journal.pcbi.1000500

**Published:** 2009-09-11

**Authors:** Balázs Ujfalussy, Tamás Kiss, Péter Érdi

**Affiliations:** 1 Department of Biophysics, KFKI Research Institute for Particle and Nuclear Physics of the Hungarian Academy of Sciences, Budapest, Hungary; 2 Center for Complex Systems Studies, Kalamazoo College, Kalamazoo, Michigan, United States of America; CNRS, France

## Abstract

A fundamental question in understanding neuronal computations is how dendritic events influence the output of the neuron. Different forms of integration of neighbouring and distributed synaptic inputs, isolated dendritic spikes and local regulation of synaptic efficacy suggest that individual dendritic branches may function as independent computational subunits. In the present paper, we study how these local computations influence the output of the neuron. Using a simple cascade model, we demonstrate that triggering somatic firing by a relatively small dendritic branch requires the amplification of local events by dendritic spiking and synaptic plasticity. The moderately branching dendritic tree of granule cells seems optimal for this computation since larger dendritic trees favor local plasticity by isolating dendritic compartments, while reliable detection of individual dendritic spikes in the soma requires a low branch number. Finally, we demonstrate that these parallel dendritic computations could contribute to the generation of multiple independent place fields of hippocampal granule cells.

## Author Summary

Neurons were originally divided into three morphologically distinct compartments: the dendrites receive the synaptic input, the soma integrates it and communicates the output of the cell to other neurons via the axon. Although several lines of evidence challenged this oversimplified view, neurons are still considered to be the basic information processing units of the nervous system as their output reflects the computations performed by the entire dendritic tree. In the present study, the authors build a simplified computational model and calculate that, in certain neurons, relatively small dendritic branches are able to independently trigger somatic firing. Therefore, in these cells, an action potential mirrors the activity of a small dendritic subunit rather than the input arriving to the whole dendritic tree. These neurons can be regarded as a network of a few independent integrator units connected to a common output unit. The authors demonstrate that a moderately branched dendritic tree of hippocampal granule cells may be optimized for these parallel computations. Finally the authors show that these parallel dendritic computations could explain some aspects of the location dependent activity of hippocampal granule cells.

## Introduction

Neurons possess highly branched, complex dendritic trees, but the relationship between the structure of the dendritic arbor and underlying neural function is poorly understood [1]. Recent studies suggest that dendritic branches form independent computational subunits: Individual branches function as single integrative compartments [2],[3], generate isolated dendritic spikes [4],[5] linking together neighbouring groups of synapses by local plasticity rules [6]–[8]. Coupling between dendritic branches and the soma is regulated in a branch-specific manner through local mechanisms [9], and the homeostatic scaling of the neurotransmitter release probability is also regulated by the local dendritic activation [10].

The computational power of active dendrites had already been demonstrated by several computational studies [11]–[16], but how local events influence the output of the neuron remained an open question. Using the cable equation [17] or compartmental modelling tools one can calculate the current or voltage attenuation between arbitrary points in a dendritic tree [14], which is in good agreement with *in vitro* recordings. However, cortical networks *in vivo* are believed to operate in a balanced state [18],[19], where the inhibitory drive is continuously adjusted such that the mean activity of the population is nearly constant [20],[21]. In this case, the firing of an individual neuron is determined, beyond its own input, by the activity distribution of the population. A simple cascade model [22] incorporating numerous dendritic compartments allowed us the statistical estimation of the activity distribution of neurons within the population. We used this model to study how localized dendritic computations influence the output of the neuron.

**Figure 1:**
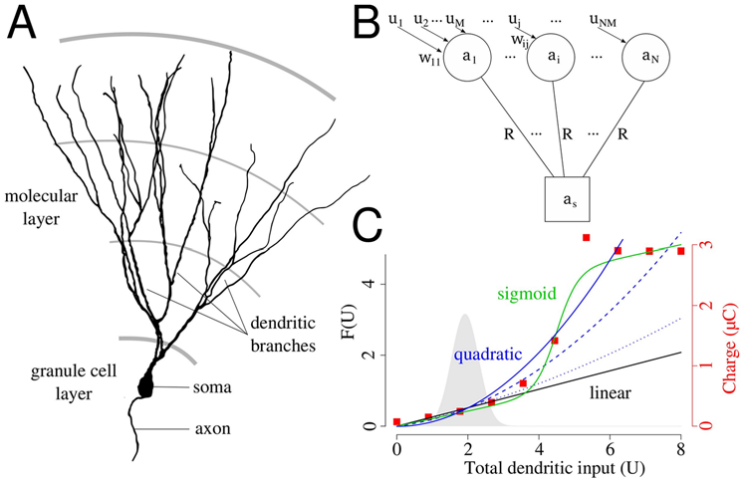
The structure of the model. (A) Anatomical reconstruction of the dendritic tree of a mature granule cell from mice. The dendritic tree is dominated by the long parallel dendritic branches in the outer two third of the molecular layer. Note the lack of basal dendrites and the apical trunk compared to a pyramidal neuron. Image courtesy of Dr. Josef Bischofberger. (B) Model for the somato-dendritic interactions in dentate granule cells. Distal dendritic compartments are represented by circles, and the soma by a square. Further details are in the text. (C) The different dendritic integration functions used in this study. Black: linear, blue: quadratic, green: sigmoid function. Red, square symbols indicate the nonlinearity of dendritic integration in a conductance based model of hippocampal granule cell (see Text S1). Dashed and dotted lines show different degrees of nonlinearity. The distribution of the total dendritic input with uniform synapses is shown in the background.

The present study focuses on hippocampal granule cells. Compared to pyramidal neurons granule cells have relatively simpler dendritic arborization: They lack the apical trunk and the basal dendrites, but are characterized by several, equivalent dendritic branches, extended into the molecular layer [23] (Figure 1A). Recordings from freely moving rats revealed that like pyramidal neurons, granule cells exhibit clear spatially selective discharge [24],[25]. However, granule cells had smaller place fields than pyramidal cells, and had multiple distinct subfields [24],[26]. It has also been recently shown that these subfields are independent, i.e., their distribution was irregular and the transformation of the environment resulted in incoherent rate change in the subfields [26]. The dendritic morphology of granule cells suggest that parallel dendritic computations could contribute to the generation of multiple, distinct subfields of these neurons.

In the present study we analyzed how synaptic input arriving to dendritic subunits influence the neuronal output. First, we introduce the model used in this study and we define statistical criteria to measure if a dendritic branch alone is able to trigger somatic spiking. We show, that generally neurons perform input strength encoding i.e., input to the whole dendritic tree but not activation of a single branch is encoded in the somatic firing. Next we demonstrate that if the local response is enhanced by active mechanisms (dendritic spiking and synaptic plasticity) then neurons switch to feature detection mode during which the firing of the neuron is usually triggered by the activation of a single dendritic branch. Furthermore we show that moderately branched dendritic tree of granule cells is optimal for this computation as large number of branches favor local plasticity by isolating dendritic compartments, while reliable detection of individual dendritic spikes in the soma requires low branch number. Dendritic branches of dentate granule cells could therefore learn different inputs; and the cell, activated through different dendritic branches, could selectively respond to distinct features (locations), participating in different memories. Finally using spatially organized input we illustrate that our model explains the multiple independent place fields of granule cells and these dendritic computations increase the pattern separation capacity of the dentate gyrus.

## Model

We set up a cascade model [22] to study the somato-dendritic interactions in neurons, that is simple enough for mathematical analysis but can be adequately fitted to experimental data. The long, parallel branches of dentate granule cells are represented by distinct compartments connected to the somatic compartment of the model (Figure 1B). The activation of the somatic 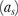 and dendritic 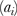 compartments are described by the following equations:
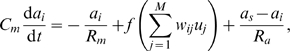
(1)

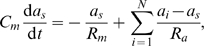
(2)where *C_m_* is the membrane capacitance, 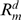 and 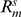 are the total dendritic and somatic membrane resistances, respectively, and 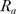 is the axial resistance between the dendritic and the somatic compartments. Each of the *N* dendritic branches are contacted by *M* presynaptic axons, *u_j_* is the firing rate of axon *j*, and *w_ij_* is the synaptic strength between the dendritic branch *i* and presynaptic axon *j* (see Methods for parameters specific to hippocampal granule cells). *f*(*U*) is the dendritic integration function that specifies the form of the local integration of synaptic inputs, and *U_i_* = Σ*_j_w_ij_u_j_* is the total synaptic input to a given branch. Because the firing rate of the presynaptic entorhinal neurons depend mostly on the location of the animal [27] we assume, that input varies slowly compared to the membrane's time constant in dentate granule cells (*τ_m_*≈37 ms, [28]). Therefore, we rewrite Equations 1–2 to their steady-state form:
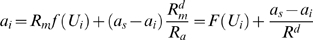
(3)

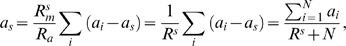
(4)where 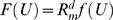 and 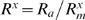 is the proportion of the axial and the membrane resistivity. In granule cells the area and the electrical resistance of the somatic membrane is similar to the membrane area and resistance of a single dendritic branch [28] (see Methods). Therefore, in the following calculations we use *R* = *R^s^* = *R^d^* to denote the electrical isolation between somatic and dendritic compartments. Three different functions were used in this study to approximate the local integration of synaptic inputs within the dendritic branches of hippocampal granule cells (Text S1, Figure 1C): a linear (*F_L_*(*U*) = 0.26*U*) and a quadratic (*F_Q_*(*U*) = 0.13*U* ^2^) function were used in the analytical calculations; and the results were also tested with a sigmoid (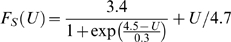) function in the Supporting Information (Text S2). We also performed some of the analytical calculations by decreasing the degree of nonlinearity, where we used *F_C_* = 0.07*U* ^2^+0.12*U* or *F_C_* = 0.02*U* ^2^+0.22*U*. Note, that the action potential generation is not incorporated in the model, and all active properties of the dendrites are modeled by the integration function *F*(*U*).

### Distribution of the Somatic Activation

Supposing that firing rates of presynaptic neurons (*u_j_*) are independent and identically distributed we assume that the total input of the dendritic branches *U_i_* = Σ*_j_w_ij_u_j_* is drawn randomly from a Gaussian distribution with mean *μ* and variance *σ* ^2^:
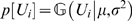
(5)where *p*[*U*] indicates a probability distribution over *U* (Figure 1C; see Eq. 17 in Methods for parameters specific to hippocampal granule cells). More specifically, 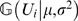 indicates the distribution of the magnitude of possible total inputs to a single dendrite over many different instances. Based on the distribution of the total input, we can compute the distribution of the somatic activation 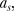 and determine the firing threshold (*β*) according to the proportion of simultaneously active cells (the sparseness of the representation, *sp*_DG_) in the DG [24]. First, we rearrange Eq. 3 using the input distribution to express the distribution of 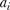:
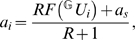
(6)where 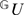 indicates that the inputs of the dendritic branches are randomly sampled from a Gaussian distribution. We substitute Eq. 6 into Eq. 4, and we get
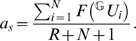
(7)We can assume again, that the inputs (*U_i_*) of the dendritic branches are independent and identically distributed variables. (Note, that while the activations 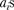 are not independent because of the back-propagation of currents from the soma, the inputs are.) If *N* is high enough, we can approximate the sum in Eq. 7 with a Gaussian distribution, and rewrite the equation:
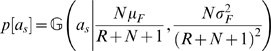
(8)where 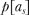 indicates a probability distribution over 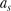, while *μ_F_* and 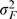 are the expected value and the variance of the dendritic integration function *F*(*U*) given the input distribution 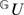:
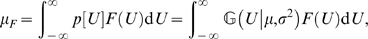
(9)

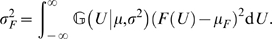
(10)We calculated the integrals 9–10 with two different forms of dendritic integration of synaptic inputs: a linear and a quadratic function (Figure 1C). The details of these calculations are in the Supporting Information (Text S4).

In this paper we do not model inhibitory neurons in the dentate gyrus, however, we assume, that they play a substantial role in continuously adjusting the firing threshold of principal neurons and regulating the activity of the network [20],[21]. As a result of this regulation always the most depolarized neurons are able to fire, and the proportion of simultaneously active neurons is characteristic for different hippocampal areas [24],[29]. Given that all neurons share a common input statistics and have similar internal dynamics, equation 8 also describes the distribution of 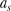 across the granule cell population at a given time. If only the most depolarized 1–5% of the population are able to fire [29], this also means that only those neurons exceed their firing threshold whose activation is within the uppermost 1–5% of the distribution described by Eq. 8. Therefore, the proportion of simultaneously active neurons within the dentate gyrus *sp_DG_* [24],[29] also determine the firing threshold *β* for granule cells.

### Criteria for Independence in the Output

We approach the dendritic independence by focusing on the statistical distributions of the input to dendritic branches, as these branches form the basic computational subunits in our model. We ask whether the input of a single branch could be sufficiently large to significantly depolarize not only the given branch but also the soma of the neuron. We defined two conditions to study whether the spiking of the neuron is caused by the activation of a single dendritic branch or by the simultaneous depolarization of multiple branches.

**Figure 2:**
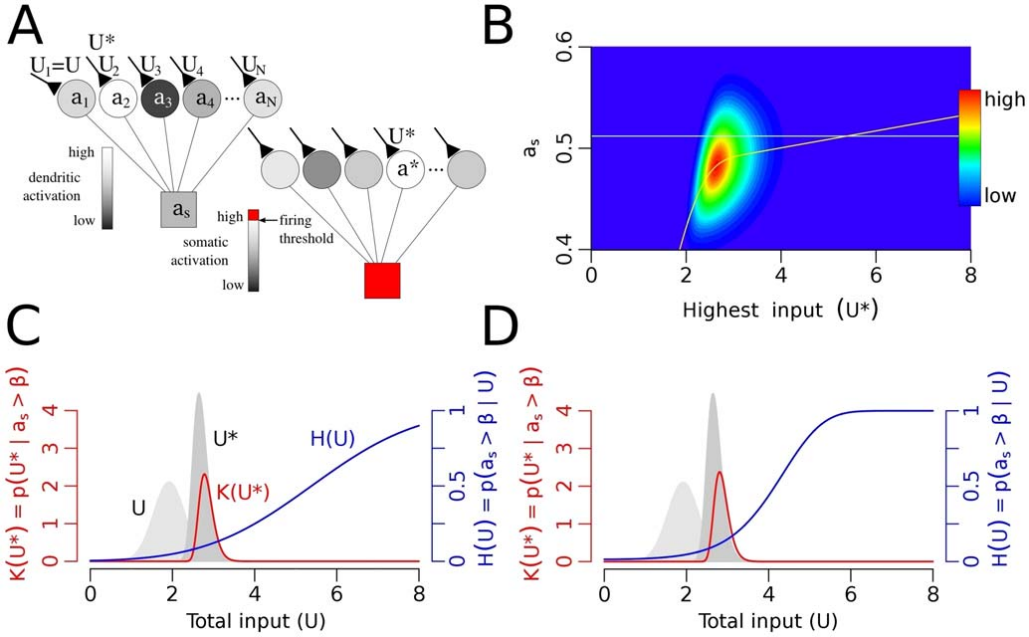
Input strength encoding with uniform synapses. (A) The figure shows two neurons (or the same neuron with two different input sets). We calculated the probability of firing (*H*(*U*)) given that one of the branches has exactly *U* synaptic input (e.g., *U*_1_ = *U*) while inputs of other branches (*U*_2_, *U*_3_, …, *U_N_*) are drawn independently from the input distribution. Second, we calculated the distribution of the maximal input (*K*(*U* ^*^)) given the depolarization of the soma exceeds the firing threshold. (B) Color coded joint probability distribution of the somatic activation and the maximal dendritic input, 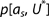 with the linear integration function. Red is maximum, dark blue is zero. The color-code emphasizes low probability events and it is not linear. The horizontal line is the firing threshold; the yellow line shows the conditional expectation of *a_s_* given *U* ^*^. If dendrites were independent high and low *U* ^*^ values could be separated by a somatic threshold of action potential generation. (C–D) Dendritic independence with linear (C) and quadratic (D) integration functions. Left axis, red: *K*(*U* ^*^), the distribution of the maximal dendritic inputs during firing. Right axis, blue: the *H*(*U*) function, which is the probability of firing given that one of the dendrites has *U* total input. The probability of triggering output by a single branch is low (*H*(*U*)<0.25) even with reasonably large input (as revealed by the low *H*(*U*) values at the probability mass of *K*(*U* ^*^)). This indicates that a single dendritic subunit is unable to reliably activate the neuron with these integration functions. Background light gray is the distribution of *U* while dark grey shows the distribution of *U* ^*^. Parameters: *R* = 0.01, *N* = 30.

First, the conditional probability 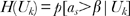 is the probability of firing given that any branch *k* has total input *U_k_* = Σ*_j_w_kj_u_j_*, while inputs to all other branches are random and independent samples from the distribution of 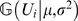 (Figure 2A). At those *U_k_* values where this probability is close to 1 the cell tends to fire when any of the dendritic branches gets that input. Second, the conditional distribution 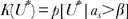 is the distribution of the synaptic input of the most active branch at the time the depolarization of the soma exceeds the firing threshold (*β*), where *U* ^*^ is the total synaptic input arriving to the most active branch (Figure 2A). *K*(*U* ^*^) can be regarded as the marginal distribution of 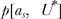 above the firing threshold (Figure 2B). The probability mass of this function shows the typical maximal input (*U* ^*^) values when the neuron fires.

These two conditions together determine whether a single branch can be sufficiently depolarized to trigger somatic spike or not. If the probability of firing is high (*H*(*U*)≈1) at typical input values (*K*(*U* ^*^)) then the firing of the cell is caused by a *single branch*. With the definition of Gasparini and Magee [30] we call this form of information processing as *independent feature detection*. On the other hand, if the firing probability is low (*H*(*U*)≪1) even if one of the branches receive extremely large input (*U* ^*^ is high) then the cell mostly fires when the *overall* dendritic activation is high, and even the most depolarized branch usually fails to make the neuron fire. We use the expression *input strength encoding* [30] to denote this second type of computation. The calculation of the two functions *H*(*U*) and *K*(*U* ^*^) is described in the Methods section.

## Results

### Constant Synaptic Weights: Input Strength Encoding

First we chose unstructured synaptic input, i.e., the firing of entorhinal neurons were independent and the strength of all synapses were equal. In this case we approximated the total synaptic input *U* to a branch with a Gaussian distribution (Eq. 5, Figure 1C). Given the input distribution we asked whether the excitation of single branches can be sufficiently large to cause significant depolarization in the soma.

The typical largest input values, indicated by the probability mass of *K*(*U* ^*^) (Figure 2C–D) are unable to sufficiently depolarize the soma and determine the neuronal output (indicated by the low *H*(*U*) values) in the case of both the linear (Figure 2C) and the quadratic (Figure 2D) integration functions. Wherever *K*(*U* ^*^) has high values, *H*(*U*) is low in both cases, which indicate, that these branches are not able to independently influence the output of the neuron. Only coactivation of several branches could make the neuron fire in this case, and the output of the neuron encodes the strength of all dendritic inputs. As *H*(*U*) converges to 1 for high input values extremely high inputs to a single dendrite could reliably trigger somatic firing. In the next sections, however, we study how synaptic plasticity selectively modifies individual synapses and contributes to the sparse occurrence of extraordinarily high input values.

### Hebbian Synapses: Feature Detection

During Hebbian learning synapses contributing to postsynaptic activation are potentiated while other synapses may experience compensatory depression [31],[32]. We simulated the learning process by showing a finite number of uncorrelated samples from the input distribution (see Methods) to the model neuron initiated with uniform synaptic weights. The synaptic weights of those dendritic branches where the activation exceeded a threshold, *β_d_* were modified according to the following Hebbian plasticity rule [33] that incorporates heterosynaptic depression [31]:
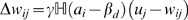
(11)where 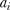 is the local dendritic activation, *u_j_* is the presynaptic firing rate and *w_ij_* is the synaptic strength. 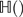 is the Heaviside function and γ<1 is a constant learning parameter. Note, that the learning rule is local to the dendritic branches: the synaptic change depends on the local activation but not on the somatic firing.

**Figure 3:**
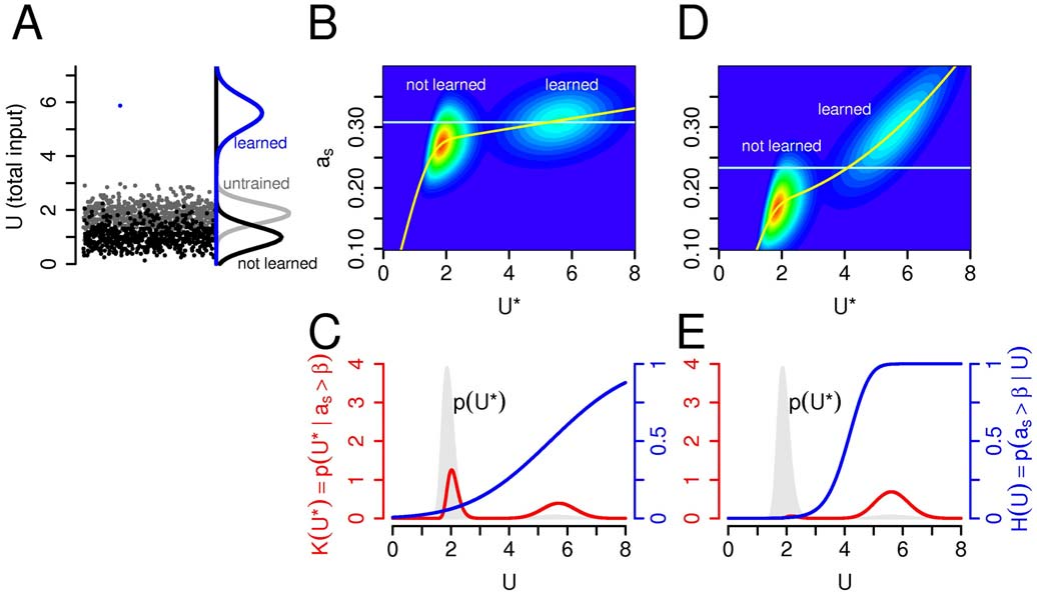
Independent feature detection with Habbian synapses. (A) Synaptic plasticity separates the inputs. Before learning the total synaptic input to a dendritic subunit come from a Gaussian distribution (600 samples are shown with grey circles) with the calculated density function shown on the right (Eq. 5). During the learning process each branch learns its largest input and the response increases to the learned input (blue circle), while it decreases to all other inputs (black circles). The black and blue Gaussian curves show the density functions for the non-learned and learned inputs, respectively (Eq. 19). (B), (D): Color-coded joint distribution of the somatic activation and the maximal dendritic input (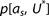) in the linear (B) and quadratic (D) case. The horizontal lines indicate the firing threshold. (C), (E): The distribution of the maximal dendritic input when the cell fires (*K*(*U* ^*^) in red) and the probability of firing with a given input (*H*(*U*) in blue). The distribution of *U* ^*^ is shown in the background. In the linear case, 50% of firing occurs when one of the branches receives its preferred input, while with quadratic integration function more than 95%. Parameters: *R* = 0.01, *N* = 30.

Next, we calculated the total input to the branches *U_i_* = Σ*_j_w_ij_u_j_* after modification of synapses (Figure 3A), and recalculated the two functions *H*(*U*) and *K*(*U* ^*^) defined previously with the new input distribution (Eq. 18). As shown on Figure 3A the total synaptic input in response to a learned pattern increases significantly after learning (compare blue and grey curves on Figure 3A), while untrained patterns generate smaller synaptic inputs (compare grey and black curves on Figure 3A). The main consequence of synaptic plasticity is that the trained patterns generate much larger local response than untrained patterns, which raise the possibility of their detection in the soma. Note, that an unspecific increase of synaptic weights would result in an upward shift of both the input distribution (Eq. 5) and the firing threshold, but would not affect the somatic detection of individual dendritic events.

The neuron is able to selectively respond to the dendritically learned patterns if a single branch, when facing with its preferred input, is able to induce significantly more depolarization at the site of the action potential initiation compared with the case when all of the branches get random, not learned input. Figure 3B–E shows the dendritic input and the activation of the soma after learning. If the maximal input *U* ^*^ is small (left bumps on Figure 3B,D) and none of the branches got its preferred input then the somatic activation is usually small. If *U* ^*^ is high (Figure 3B,D; right bumps), which means that one of the branches receives its preferred input pattern, then the somatic activation is increased. The increase of the somatic activation with learned input is only moderate in the linear case (Figure 3B,C) resulting in an incomplete separation of learned and not learned inputs by the somatic firing threshold. However, if synaptic inputs are supra-linearly (quadratically) integrated within the dendritic branches, efficient separation is possible: the probability that the presentation of a learned pattern elicits subthreshold somatic response, called *dendritic spike detection probability* was over 95% (Figure 3D,E). In this case the output of the neuron encodes whether or not one of the stored features was present in the neuron's input and not simply the strength of the total input arriving to the whole dendritic tree. In other words, if dendritic nonlinearity enhance the response of a given branch to its preferred input, then this branch alone is able to trigger somatic spiking. In the following sections we use the term *dendritic spiking* to refer to these supra-linear dendritic events. Although there is no data available on the synaptic induction of local dendritic spiking in hippocampal granule cells, voltage dependent Ca^2+^ currents are present in the membrane of granule cells [34],[35] and whole-cell recordings from these neurons suggest that T-type Ca^2+^ channels can generate dendritic action potentials at least in young neurons [36] or under hyper-excitable conditions [34],[37].

### Independent Learning in Isolated Branches

**Figure 4:**
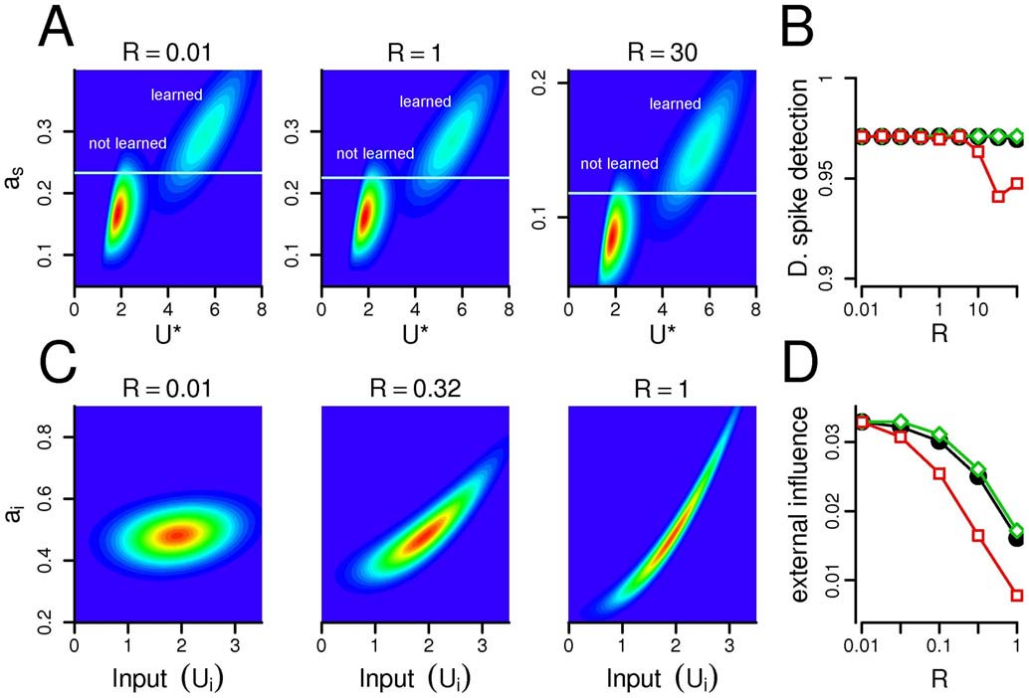
Changing the resistance influence the isolation of compartments, but not the detection of dendritic spikes. (A) The joint distribution of the somatic activation *a_s_* and the maximal dendritic input *U* ^*^ with different resistances. (B) The probability of detecting a dendritic spike remains constant even if the resistance changes 2–3 order of magnitude. Black circles: both somatic and dendritic resistances are altered; green diamonds: only resistance of the dendritic membrane (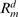) is changed while 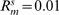; red squares: somatic membrane resistance (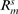) is changed, 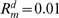. Although the distribution of the somatic activation scales with the resistance (see panel A and Eq. 7), the detection probability of a single dendritic event remains relatively constant. (C) The joint distribution of the activation of a dendritic branch 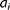 and its own input *U_i_*. When the resistance is low (left), the local activation depends only slightly on the input. Conversely, if the resistance is higher (right) the local input has substantial impact on the activation of the branch. (D) The external influence decreases as the resistance increases. Symbols are the same as on panel B. Note, that decreasing the resistance of the perisomatic membrane (red squares) is the most efficient in separating the dendritic compartments (recall, that 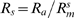). *N* = 30.

Next, we explored how the independent feature detection ability of the model depends on the resistance between the somatic and dendritic compartments with nonlinear dendritic integration. In the passive cable model of dendritic trees the space constant of the membrane *λ_m_*≈(*R_m_*/*R_i_*)^1/2^ plays a substantial role in determining the voltage attenuation among two sites. Consequently, an increase in the intracellular resistivity *R_i_* or a similar decrease in the membrane resistance *R_m_* will contribute to the separation of dendritic subunits by decreasing the membrane's space constant *λ_m_*. In the present study we used the inverse of the space constant *R*≈*R_i_*/*R_m_* to characterize the degree of electrical resistivity between the somatic and dendritic compartments. Indeed, an increased resistivity (*R*) between the compartments (smaller space constant) induced larger degree of electrical isolation as the somatic response to the same amount of dendritically applied current decreased (compare Figure 4A left and right panels). However, this isolation did not modify the dendritic spike detection probability in the soma: Large dendritic spikes localized to a single compartment could be reliably separated from subthreshold events with a somatic firing threshold at a large range of resistances *R* (Figure 4A–B). This was also true for the selective alternation of the somatic or the dendritic membrane resistance (Figure 4B).

On the other hand, the resistance parameter had a substantial impact on the isolation of different dendritic compartments which might be necessary for the independence of synaptic plasticity. To measure the isolation of the dendritic subunits we calculated the influence of other compartments on the activation of a given branch (external influence) quantified by the standard deviation of 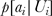. Figure 4C shows the activation of a dendritic branch in the function of its input at different *R* values. If the resistance is small (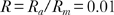, Figure 4C, left), then the local activation depends only slightly on the local input and the external influence is high (Figure 4D). In this case the local input spread out to the entire dendritic tree and activates similarly all branches. On the other hand, if the resistance is high (*R* = 1, Figure 4C, right) then the external influence is small, and the depolarization of a dendritic branch depends mostly on the local input. Interestingly, decreasing the resistance of the perisomatic membrane (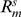) alone was more efficient in separating the dendritic subunits than decreasing the resistance of the dendritic membrane or both (Figure 4D). The extensive GABAergic [38],[39] and glutamatergic [40] innervation of the proximal dendritic and perisomatic region of granule cells may therefore contribute significantly to the isolation of the dendritic compartments.

**Figure 5:**
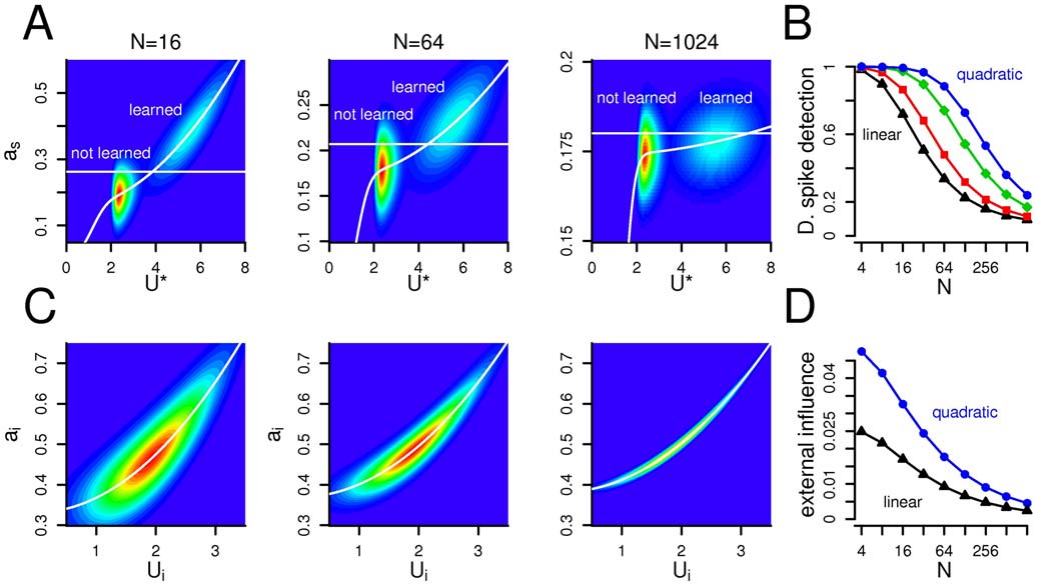
Moderate number of branches allows the isolation of subunits and the detection of dendritic spikes. (A) The joint distribution of the somatic activation as and the maximal dendritic input *U* ^*^. As the number of branches grow (from left to right), the somatic depolarization caused by a dendritic spike in a single compartment decreases gradually. Consequently, if *N* is high, than the somatic threshold (horizontal line) can not separate small and large dendritic events. (B) The probability of detecting a dendritic spike decreases as the number of dendritic subunits increases. Different colors indicate different degrees of nonlinearity (blue circles: quadratic; black triangles: linear integration function). (C) The joint distribution of the activation of a dendritic branch 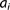 and its own input *U_i_*. Increasing the number of compartments decrease the variance of the distribution and the impact of other branches. (D) The external influence decreases with the number of branches both with linear (triangles) and quadratic (circles) integration function. *R* = 0.3.

The impact of a single branch on the somatic activation, and also the coupling between dendritic branches may depend highly on the structure of the dendritic tree. Therefore we varied the number of dendritic subunits, *N*, and calculated the probability of detecting dendritic spikes in the soma and the external influence on the dendritic subunits (Figure 5). The probability of detecting a dendritic spike in the soma decreased gradually after a few (*N*≈30) number of branches from 1 to 0.3 (*N*≈1000, Figure 5A–B). If the number of branches was low, then the effect of a single branch on the soma was relatively high, and the somatic detection of single dendritic events was reliable. Conversely, one out of hundreds of branches had relatively low impact on the neuron's output even if the local depolarization was significant.

The electrical coupling between the dendritic subunits characterized by the external influence on the local activation also decreased with the number of branches, (Figure 5C–D). In the model the branches are connected through the somatic compartment, and because the variance of the somatic activation decreases if *N* increases (Eq. 8), the external influence will also decrease. However, in a complex dendritic tree containing higher number of subunits the branches are electronically more isolated which is required for local plasticity. To keep the probability of dendritic spike detection high and the dendritic coupling low at the same time, the number of branches should therefore be as high as possible, but not higher than *N*≈60.

As we showed on Figure 4, the dendritic coupling depends on the resistance *R*, as high resistance separates better the subunits. Therefore we conclude, that a medium number of branches with relatively high resistance is ideal for parallel dendritic computations. The optimal number of dendritic subunits, however, depends on the size of the dendritic event determined by the local integration of the synaptic inputs (Figure 5B). Appropriate detection of dendritic responses to learned patterns with linear integration is possible only in very small dendritic trees, whereas supra-linear integration allows the detection of individual dendritic events also in a larger dendritic arbor. Nonlinear integration by dendritic spiking therefore permits the neuron to selectively respond to a larger number of distinct input pattern.

### Verification of the Model with Location Dependent Input

During the calculation above we assumed, that the activity of the presynaptic neurons are independent and that the samples from the distribution are uncorrelated. It is known, however, that the firing of entorhinal neurons are not independent: At least half of layer II cells in the medial entorhinal cortex (EC) are grid cells, whose firing depend mostly on the position of the animal [27]. Moreover, in reality animals do not face with discrete uncorrelated samples, but they experience the continuous change of their environment which is mirrored by the activity of the entorhinal neurons. In order to test our model under more realistic conditions, we simulated the activity of the rodent's EC during exploratory behavior as input to our modeled granule cell. The EC consisted of two neuron population: A population of grid cells (1000 neurons, 5 spacing, 5 orientations) representing a path integrator system [41] and a population of visual cells (1200 units), representing highly processed sensory information available in the EC [42]. In these simulations we used the *Webots* mobile robot simulator [43].

The firing statistics of the entorhinal neurons was the same as used in the analytical calculation except that the activity of the neurons was location dependent. Moreover, as we simulated the trajectory of the rat during continuous foraging for randomly tossed food pellets [26] the subsequent input patterns were highly correlated. We simulated a single granule cell with *N* = 20 dendritic branches each of them receiving a total number of *M* = 100 synaptic contacts from entorhinal neurons. The resistance was *R* = 1, we used the quadratic integration function and the neuron was tested in 5 different environments. During the 5 min. learning period (while 2000 spatial locations was sampled with an average running speed of 0.22 m/s) 0–8 branches learned usually at different spatial locations in each of the 5 environments. In most of the time synaptic plasticity in different branches occurred at different places, therefore the subunits were able to learn independently. Moreover, learning occurred only in naive branches, i.e., each branch learned only in one environment at a specific location and synapses of trained branches did not engage in learning at a different location. After the training period the synaptic weights of those branches that were subthreshold for synaptic plasticity (*β_d_* = 1.11) in all environments were scaled down manually.

**Figure 6:**
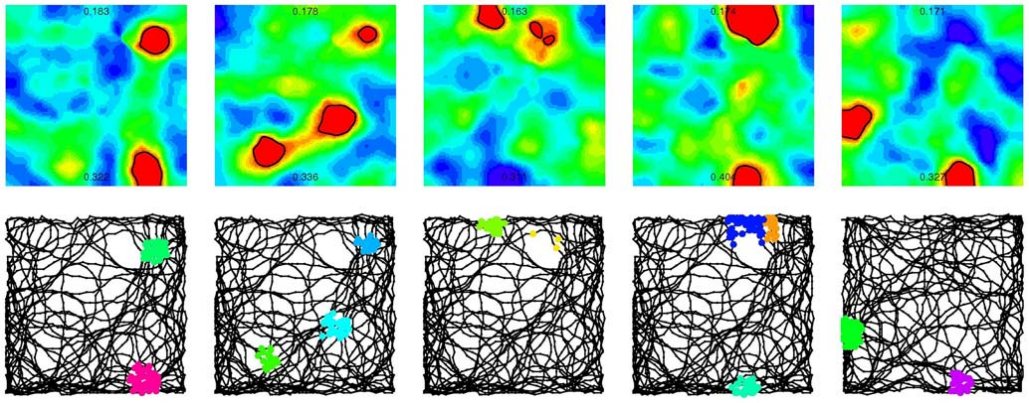
Location dependent input and parallel dendritic computations generate multiple place fields. The behavior of the same granule cell in five different environments (columns). Upper row: color-coded maps (“ratemaps”) show the somatic activation on the 1×1 meter large maze. Red: high activation (spiking), blue: silent. The highest and the lowest value of the somatic activation is indicated on each ratemap. The places where the activation exceed the threshold (“place fields”) are surrounded by black lines. We used the same, linear color-code in all panels. Lower row: the track of the robot and the location of the dendritic spikes. Dendritic place fields of different branches of the same neuron are marked by different colors. Somatic firing usually coincide with the activation of single dendritic branches.

Next we studied the spatial activity pattern of the somatic and dendritic compartments while the robot was moving on a different track in the same environments. The dendritic branches responded with high activation (“dendritic spikes”) to subsequent visit of places close to their preferred locations leading to the formation of dendritic place fields (Figure 6). Moreover, since the activation of the soma was substantially increased in each of these dendritic place fields, the neuron had a multi-peaked activity map in several environments (Figure 6).

**Figure 7:**
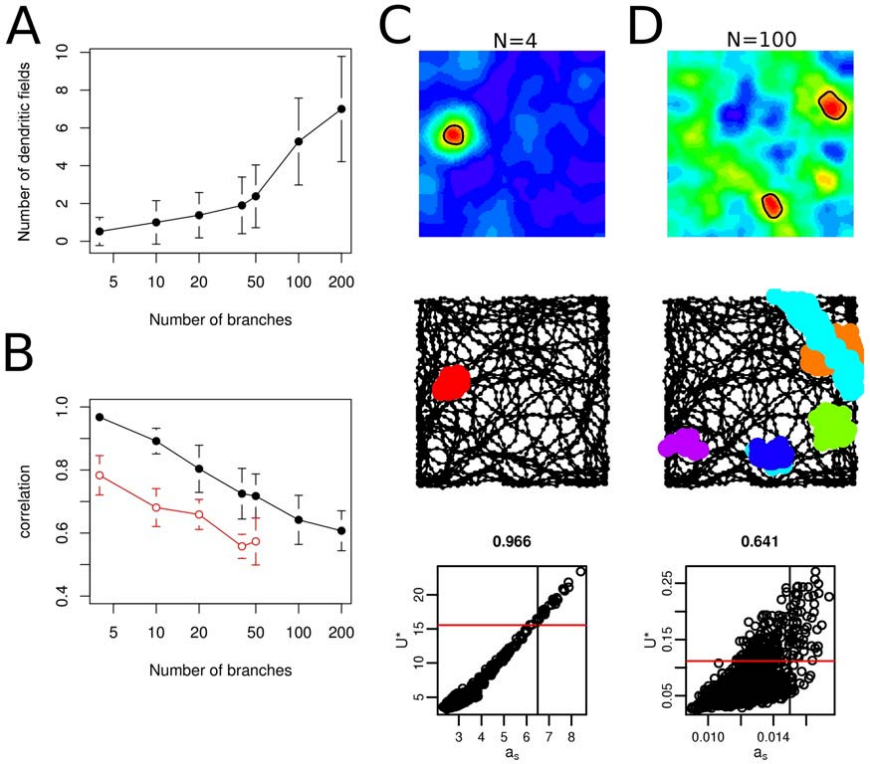
Spatial firing patterns with different number of dendritic subunits. (A) The number of dendritic place fields increases with the number of subunits although the probability of learning were kept constant. (B) The correlation between the somatic activity (*a_s_*) and the maximal input (*U* ^*^) decreases if the number of dendritic subunits increases. Open circles: correlation in the absence of dendritic spikes. Error bars on A and B show the standard deviation of 50 trials in 5 different environment. (C–D) Spatial firing patterns with *N* = 4 (C) and *N* = 100 (D) dendritic subunits. Upper and middle row is the same as on Figure 6. Lower row: scatter plot showing the joint distribution of the somatic activation *a_s_* and the maximal dendritic input *U* ^*^. Threshold for synaptic plasticity in the branches (horizontal line) and somatic firing (vertical line) are indicated. The correlation between the two variables is shown above the panels. If the neuron has a small number of dendritic subunits than the number of dendritic fields is small (A, C) but they propagate efficiently to the soma. Conversely, a neuron with a large number of dendritic subunits might have numerous dendritic fields, but the individual dendritic spikes have a little impact on the somatic activation.

Finally we explored the effect of the size of the dendritic tree on the spatial firing pattern of the neuron (Figure 7). If there were only a few functional dendritic subunit than the neuron obviously had a small number of dendritic place fields (Figure 7A), but the individual branches had strong influence on the somatic activity. Therefore the correlation between the somatic activation *a_s_* and the maximal dendritic input *U* ^*^ was high (Figure 7B,C), as predicted by the analytical calculations. On the other hand, in neurons with large number of dendritic subunits there were more dendritic place fields (Figure 7A), but a single branch had only a little impact on the activity of the neuron (Figure 7D). Accordingly, the correlation between the maximal dendritic input and somatic activation was reduced (Figure 7B). In these cases the cell fired when the overall excitation was high or when more than one branch were simultaneously excited. Therefore, the moderately branching dendritic tree of granule cells seems optimal for parallel dendritic computations since extensive branching inhibits the detection of individual dendritic events.

We conclude, that clustered plasticity together with dendritic spiking may be an adequate cellular mechanism to explain the generation of multiple place fields in the DG [24],[26].

## Discussion

In the present paper we set up a statistical criteria to determine the effect of single dendritic events on the output of the neuron. Using this criteria we have shown that by supra-linear dendritic integration, given that branches have learned different input patterns, individual dendritic branches are able to trigger somatic firing. Next we have shown that high resistivity and large number of branches supports the segregation of dendritic subunits required for local plasticity. On the other hand, a single branch has a substantial effect on the output of the neuron only if the number of branches is sufficiently low. Finally using spatially organized input we have demonstrated that parallel computational subunits explain multiple, independent place fields of hippocampal granule cells.

### Dendritic Spiking

Dendritically generated spikes mediated by voltage-gated Na^+^ [3] and/or Ca^2+^ channels [44] as well as glutamate-activated N-methyl-D-aspartate (NMDA) channels [45] have been described in a variety of neurons (for a review see [46] or [47]) including hippocampal granule cells [34]–[37]. We used a quadratic integration function in order to analytically model supra-linear dendritic integration [15] which differs from the sigmoid form of nonlinearity realized by dendritic spiking (Text S1, [3],[4],[45]). We believe, however, that at this level of abstraction the exact form of nonlinearity does not affect our results: As that is the difference between the dendritic responses to learned and not learned patterns that influence the somatic detection of dendritic events, a sigmoid integration function give qualitatively similar results (Text S2). Moreover, we studied only passive interactions between individual dendritic events as the effect of voltage and calcium dependent currents (including A-type and Ca^2+^-dependent potassium [48] and the H-current [49]) regulating the propagation of dendritic spikes were not included in the model. Future studies using a compartmental model equipped with dendritic spiking could support our results and clarify further details.

Our analysis has revealed that a moderately branched dendritic tree is optimal for the independent branches model, and we have shown that this mechanism could contribute to the spatial firing properties of granule cells in the DG. The dendritic tree of cerebellar Purkinje cells as well as the apical dendrites of hippocampal and neocortical pyramidal cells is typically larger, and more ramifying [50]. Their morphology is suitable for local plasticity within single branches [6],[8], and although it seems that individual branches may function as single integrative compartments [3],[4],[51],[52], dendritic spikes localized to these compartments fail to propagate to the soma and directly influence the neuron's output [53]. Larger dendritic events, active spread of dendritic spikes towards the soma or interactions among dendritic subunits could contribute to the generation of somatic action potentials in this case. The dendritic tree of pyramidal neurons is, however, far more complex than that of granule cells: it has several morphological and functional subregions with different afferent inputs and membrane excitability [50]. Understanding how their spatial firing characteristics arise from their cellular properties would require at least a different model structure and is beyond the scope of this paper.

Whether individual dendritic events influence the output of the neuron depends - beyond the structure of the dendritic tree - on the size and the frequency of the large dendritic events and the output sparsity. The size of the events depends on the exact form of the dendritic integration function and the plasticity rule while the input statistics determine the frequency of such events. We have shown that given the sparseness of the output, sufficiently large, localized dendritic events arriving with appropriate frequency are able to separately determine the output of the neuron. Whether a local event is sufficiently large depends on the geometry of the dendritic tree: A smaller event may be sufficient if there are only a few subunits, or if the events actively propagate to a large part of the entire dendritic tree (e.g, the apical tuft in pyramidal neurons, [54]). Conversely, in neurons such as cerebellar Purkinje cells with large, ramifying dendritic tree, where individual events are localized to small branches, very large dendritic spikes would be required to influence the output. Indeed, detailed compartmental modelling of dendritic morphology revealed that the forward propagation of the action potential initiated in the apical trunk of pyramidal neurons was very effective, while in Purkinje cells dendritic action potentials were rapidly attenuated [53].

### Isolation of Branches

Clustered plasticity allows the neuron to simultaneously learn several different patterns but requires the electrical and/or biochemical isolation of the dendritic compartments [47],[55]. However, the intracellular resistance (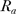) in dentate granule cells is relatively low and granule cells are usually regarded as electrically compact neurons [28]. Indeed, signal propagation from somata into dendrites *in vitro* is more efficient in granule cells compared with CA1 pyramidal cells and distal synaptic inputs from entorhinal fibers can efficiently depolarize the somatic membrane of granule cells [28]. However, *in vitro* studies do not take into account that neurons are embedded in a network of spontaneously active cells. As thousands of synapses bombard the dendritic tree *in vivo*, the dendritic membrane becomes “leakier” and, consequently, the membrane's space constant decreases significantly [56]. Moreover perisomatic inhibition [57] and feed-back excitation (via hilar mossy cells [40]) further decrease the resistance of the proximal membrane contributing to the separation of the somatic and dendritic compartments [54],[58]. More specifically, we predict, that the membrane resistance of granule cells is considerably smaller at the perisomatic region than in the distal dendrites. Indeed, computational studies predict a 7–30 fold increase in the somatic leak conductance due to the synaptic background activity [59]. On the other hand, large space constant at long terminal branches facilitate interactions among synapses distributed on the same branch. Therefore the long dendritic branches of dentate granule cells may act as single integrative computational subunits, separated from each other by the perisomatic region of the cell. Furthermore, in the present paper we used steady-state approximations and we neglected temporal characteristics of the input and the integration. For rapidly varying inputs the coupling between dendritic sites and the soma is much smaller than for slowly varying currents since the distributed capacitance throughout the tree will absorb the charge before it reaches the soma [14]. Therefore dendritic compartments in a passive tree are more isolated for transient events such as dendritic spikes than for steady-state current. Finally, biochemical compartmentalization is likely to play a substantial role in the cooperative induction of LTP in both hippocampal [60] and neocortical neurons [7].

If, on the other hand, dendritic branches are not isolated during the learning process and synapses across the whole dendritic tree are modified simultaneously then different dendritic branches will be sensitive for different component (modalities) of the same episode. A new episode with partial overlap with the previously learned one may trigger dendritic spiking in the corresponding dendritic branch. As the somatic detection probability of dendritic spikes does not depend on the degree of electrical isolation (Figure 4), individual branches trigger somatic spiking, and, in this way the dentate gyrus contributes to the associative recall of the previously encoded episode in the hippocampus.

### Synaptic Plasticity

Since the first description of LTP at perforant path - granule cell synapses [61] synaptic plasticity has become widely accepted as the physiological basis of memory [62]. As Hebbian plasticity is intrinsically unstable, simply because it is a positive feed-back mechanism multiple stability-promoting mechanisms have been proposed, including heterosynaptic depression [31],[63]. Indeed, in the present model synaptic plasticity results in an average decrease of synaptic strengths (Figure 3A), which have several functional consequences: First, as the dendritic response to untrained patterns and likewise the baseline activation of the cell decreases during training, the somatic detection of individual, large dendritic events becomes easier. Consequently, feature detection is less efficient in semi-trained neurons where synaptic weights at only a part of the dendritic tree has already been modified due to the learning precess. Therefore, in this model, appropriate training of each dendritic branch is required for proper functioning. Second, increased excitability stimulates learning in naive branches, while decreased responsiveness of previously trained branches prevents overlearning. Indeed, newly generated granule cells are more excitable than the neighboring old neurons [36], and they are preferentially incorporated into functional networks in the dentate gyrus during acquisition of new memories [64].

One of the most interesting prediction of the present model is how the number of presynaptic spikes required for the postsynaptic induction of dendritic spiking changes during the course of learning. We can calculate this by dividing the total input *U* needed for dendritic spiking with the mean synaptic weight parameter (*μ_w_*) before and after learning. Our model predicts, that while in young neurons the simultaneous occurrence of ≈70–80 presynaptic spikes (randomly distributed across the presynaptic neurons) would trigger a postsynaptic dendritic spike, after learning (i.e., in matured neurons)≈130–160 would be required.

A recent study showed that the homeostatic regulation of the neurotransmitter release probability at neighbouring synapses depends on the local dendritic activity [10]: Increased dendritic depolarization elicits a local homeostatic decrease in the release probability and vice versa. This mechanism may also prevent overlearning in trained branches where dendritic spikes has sufficiently high rate by reducing the excitability of that branch. On the other hand the same mechanism may stimulate learning new patterns in naive or disused branches where dendritic spikes are not present. One of the key elements of our model was the local nature of the synaptic plasticity, i.e., the change of the synaptic weights was controlled by the local dendritic but not the somatic activity [6]–[8]. Specifically, in hippocampal granule cells the induction of LTP was shown to be independent of the discharge of the neurons during the high-frequency stimulation [65]. Our model predicts that, if the postsynaptic signal for synaptic plasticity is localized to individual dendritic branches than, due to the associative nature of the LTP, the synapses from entorhinal cells with overlapping firing become potentiated. If LTP is accompanied by structural remodeling, than the entorhinal neurons with overlapping place fields project to the same dendritic branches of granule cells as also proposed by Hayman and Jeffery (2008) [66].

The variation in the strength of perforant path-granule cell synapses was found to be critical in the generation of multiple place fields in a recent modelling study [67]. This heterogeneity caused a greater average synaptic excitation in a fraction of granule cells. This extra excitation therefore selects the subpopulation of neurons active within a given environment similar to the proposed role of contextual inputs in the model of Si and Treves [68]. One possible source of synaptic heterogeneity is synaptic plasticity [69] which was also crucial in the present model to amplify the local responses to learned patterns.

### Hippocampal Circuitry

Hippocampal granule cells receive afferent fibers from the medial and the lateral portion of the entorhinal cortex, and these two pathways differ both in their pattern of termination [70],[71] and information content [72]. Fibers originating in the lateral EC display weak spatial selectivity and terminate on the most distal branches of granule cells, while medial entorhinal neurons innervate the middle third of their dendritic tree and show strong spatial selectivity [72],[73]. It has been recently suggested by modelling studies [66],[68] that inputs originating from the lateral EC conveys contextual information to granule cells. In these models the contextual input select a subpopulation of neurons (or dendritic branches in [66]) that can be activated within the given context (environment) while medial entorhinal fibers determine the exact location of the place fields. The selection of a subpopulation by contextual inputs can also contribute to the multiple firing fields of granule cells by reducing the number of available neurons within the given environment [67],[68]. However, the spatial distribution of the individual place fields become regular (grid-like) if the multiple firing peaks are the consequence of an incomplete competition between neurons, especially if the input grid cells are organized into a finite number of ensembles [74],[75].

In the present paper we have shown that synapses, irrespective of their origin, arriving at different branches of hippocampal granule cells can be modified at different spatial locations. We have also shown, that in granule cells each dendritic branch is able to activate the neuron, therefore each subfield on the cell's multi-peaked activity map corresponds to a dendritic place field. The segregation of contextual and positional information could explain the sensitivity of the subfields to contextual manipulations [26],[66] and is consistent with the role of DG in context discrimination [76].

Along with the laminar organization of excitatory input, different interneurons innervate different dendritic domains of granule cells [39],[77]. It appears, that distinct types of interneurons have evolved to selectively and locally modulate the computations performed by the postsynaptic membrane [57],[78]. According to our model, basket and axo-axonic cells may continually adjust the inhibitory drive such that the mean activity of the population remains nearly constant; HICAP cells, targeting the proximal dendritic domain of granule cells together with the excitatory mossy cells [40] may increase electrical isolation of distal dendritic regions by raising the conductance of the proximal membrane; whereas MOPP and HIPP cells associated with the entorhinal afferents may contribute to the de-inactivation of calcium channels required to dendritic spiking by providing rhythmic hyperpolarization to distal dendritic branches.

Hippocampal interneurons have also a substantial role in shaping the temporal dynamics of the network [78]. The firing of neurons in the hippocampal formation is strongly modulated by the theta rhythm [25],[79],[80] which is a prominent, large amplitude field potential oscillation in the rodent hippocampus during exploratory behavior [81]. The relative synchronization of presynaptic spikes by the theta rhythm allows the temporal integration of their postsynaptic potentials despite the relatively small time constant of granule cells' membrane [28]. Moreover, the synchronization of synaptic inputs can also influence the form of dendritic integration by switching from linear to nonlinear integration [30]. Extending the present model with temporal dynamics could be an exciting direction for future research.

### Functional Consequences

What is the additional computational power gained from the present model? We argue, that smaller and uncorrelated place fields may help pattern separation in the dentate gyrus. Theoretical considerations suggest that the DG helps the hippocampal storage of new episodes by producing sparse representations via competitive learning [82],[83]. It was demonstrated by modelling studies that competitive learning on spatially organized input results in the formation of place fields [68],[74],[84],[85] that is a sparse and orthogonal representation of the input space. In the present paper we proposed that parallel dendritic computations explain the formation of multiple, independent place fields of hippocampal granule cells even within a relatively small environment [24],[26].

Pattern separation by the DG can be more efficient if granule cells have multiple, irregularly placed fields and the individual fields are smaller. The neural representation of neighbouring locations is more similar if neurons have one, larger field than if they have several but smaller fields (Text S3). In our model the place fields of a dendritic branches are analogous to the to the single place field of an electrically compact neuron. The multi-peaked somatic firing of the granule cells mirrors the several *dendritic fields* of the same neuron. We argue, that if the size of the somatic firing fields is limited by competition between simultaneously active neurons [86], then the place fields of granule cells could be smaller than the corresponding dendritic fields. If the individual place fields of granule cells become smaller, than the neural representation of adjacent places becomes less correlated which further increase the pattern separation ability of the DG. Therefore independent dendritic subunits increase the computational power of the DG while keeping the number of cells and their sparsity constant. Moreover, clustering of different inputs into different dendritic domains could explain the remapping of hippocampal place cells under several experimental conditions [26],[66].

The impact of both dendritic nonlinearity and clustered plasticity on the computational power of neurons was rarely addressed by modeling studies. Poirazi and Mel [16] predicted, that nonlinear dendritic integration with local (structural) plasticity rule increase the representational capacity of neural tissue. They showed on binary input, that the number of attainable input-output functions (representational capacity) is maximal if the neuron has many, relatively short branches, and the performance of the model in a linear classification task correlates remarkably well with the logarithm of representational capacity. However, in order to approach the combinatorial bound of the representational capacity in a neural tissue and to amplify slight differences in the input extremely large subunit nonlinearity was required (they used *F*(*U*) = *U* ^10^). In the present study we showed that a moderate increase in the memory-capacity can be achieved with local, Hebbian learning rule and slightly supra-linear dendritic integration. We emphasized that under certain conditions a single branch is able to evoke somatic output. However, if the amplitude of the individual events is smaller, a larger spatial extent involving the depolarization of additional branches will be required to trigger output spiking. This mechanism could induce a combinatorial increase in the representational capacity as shown by [16].

According to our model hippocampal granule cells can be regarded as a two layer neural network of abstract integrate and fire elements: In the first layer corresponding to the terminal branches the units integrate separately their inputs and they innervate a common output unit (second layer, the somatic compartment) that implements a logical OR computation. The idea that a dendritic tree may perform logical computations was originally proposed by [87] to explain directional selectivity of retinal ganglion cells. Shepherd and Brayton [88] further elaborated this approach but instead of branches they used dendritic spines as basic computational subunits. Our approach is more similar to how Poirazi et al. [89] describe hippocampal pyramidal cells, however, in that model the output unit performs (nonlinear) summation prior to final thresholding. Another similar model was proposed by Gasparini and Magee [30], in a paper where they showed that the apical trunk of hippocampal pyramidal neurons integrate spatially clustered and synchronously arriving synaptic inputs nonlinearly, whereas distributed or asynchronous inputs are linearly integrated. They suggest that processing in the nonlinear mode could functionally separate the dendritic arbor into a large number of independent nonlinear computational units, each sending its own output to the soma. In the present paper, we showed that a single computational units is powerful enough to determine the output of the neuron only if there are not too much similar units (N<100) and if the local integration is sufficiently nonlinear.

A similar picture emerged form a recent series of *in vitro* experiments performed on the basal dendrites of neocortical pyramidal neurons: These branches behave as independent computational subunits as nearby inputs on the same branch summed sigmoidally due to the presence of local NMDA spikes [2],[45],[90] and synaptic plasticity required the pairing of local NMDA spikes with biochemical signals [7]. Moreover, an NMDA-spike localized to a single basal dendrite could efficiently induce somatic UP-state like depolarization accompanied by bursts of action potentials [5]. These results suggest that our model describes remarkably well the neuronal computations performed by the basal dendritic tree of pyramidal neurons.

### Experimental Predictions

Although we tried to fit our model to the available experimental data we had to make some assumptions regarding the integration of neighbouring inputs in dentate granule cells. Moreover, based on the model described in the present paper we make some explicit predictions. Both the assumptions and the predictions of our model should be tested experimentally.

1. Large synaptic inputs induce a nonlinear increase in the activation of the terminal branches of dentate granule cells. The form of the dendritic integration function in granule cells, like in pyramidal neurons [3],[44],[45], could be determined by patch-clamp recordings from hippocampal slices.
2. Dendritic spiking in individual branches of young versus old granule cells can be triggered by at least 70–80 versus 130–160 simultaneous presynaptic spikes, respectively. The unitary EPSCs and the failure rates [91] at the perforant path synapses should be determined and compared with the current required for the initiation of dendritic spiking in young and old granule cells.
3. Individual dendritic branches of dentate granule cells function as a single integrative compartment. *In vitro* experiments using two-photon imaging and glutamate uncaging [2],[3],[5] could be used to test this prediction.
4. The dendritic branches of dentate granule cells are isolated from each other - at least during training - by the low input resistance of the perisomatic region. Simultaneous recording from the soma and different branches [5] together with perisomatic conductance injection [54] or detailed compartmental model paying attention to interneuronal firing rates and anatomical connectivity could determine the degree of isolation between the individual branches.
5. As the result of the local Hebbian learning rule, we predict that the presynaptic entorhinal cells with overlapping firing project to the same dendritic branches of the granule cells.
6. Different place fields of dentate granule cells are caused by excitation through different dendritic branches. This prediction could be tested *in vivo* using high resolution electrode arrays and single-cell current source density analysis [86],[92] or fiberoptic system combined with fluorescent dyes [93].

## Methods

### Estimation of the Membrane Parameters for Hippocampal Granule Cells

We used the data from [28] to estimate the passive membrane parameters of the granule cells the DG (Table 1). First we computed the membrane area of a single branch (*A_dend_*) falling into the perforant path termination zone (the outer two third of the dendritic tree):
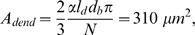
(12)where *l_d_* is the total length of the dendritic tree, *d_b_* = 1.1 µm is the average diameter of a single branch, N is the number of branches and *α* = 1.9 is a correction factor for the membrane area of dendritic spines.

**Table 1:** Membrane parameters of hippocampal granule cells.

Parameter	Description	Value
*N*	Number of branches	32±3
*M*	Num. of presynaptic terminals/branches	100
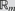	Membrane resistance (kΩcm^2^)	38±2.3
*R_i_*	Intracellular resistivity (Ωcm)	194±24
*l_d_*	Total length of dendritic tree (µm)	2264±133
*d_b_*	diameter of branches: proximal, distal (µm)	1.51±0.11, 0.73±0.04
*d_s_*	diameter of the soma (µm)	10
*a*	Surface area (1000 µm^2^)	13.3±0.9
*α*	The increase of the surface area by dendritic spines (%)	190

Electrophysiological data from Schmidt-Hieber et al. (2007) used to estimate the parameters of the model.

Similarly, the area of the somatic compartment (*A_soma_*), assuming a sphere with diameter *d_s_*:
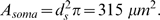
(13)The area of the cross section of a single branch is 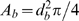, and the length of the proximal third of the branches, that do not receive input from the entorhinal cortex is *l_ds_* = 50 µm. Finally, we estimate the parameters in Eqs. 1–2:
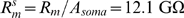
(14)

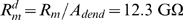
(15)

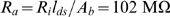
(16)where *R_m_* and *R_i_* are the membrane resistance and the intracellular resistivity, respectively. As the somatic and the dendritic membrane area (and hence the resistance) were similar, we used that 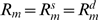. The parameter *R* used in our calculations was *R* = *R_a_*/*R_m_*0.01 for a passive granule cell in the DG. Note that due to the synaptic conductances activated *in vivo* the membrane resistances of functioning granule cells are certainly lower than its *in vitro* estimates [59].

### Estimation of the Synaptic Input

A single dentate granule cell receive synaptic input from *n*_EC–DG_≈2500–4000 entorhinal layer II cells distributed on *N*≈25–40 branches, whereas a single branch receives *M*≈100 synapses in the rat's hippocampus [28]. According to Amaral and Lavenex [71], there are *n*_DG_≈1.2 · 10^6^ granule cells in the rat's DG, and *n*_EC_≈0.11 · 10^6^ projection cells in the layer II of the entorhinal cortex. It is known, that a given location in the hippocampus may receive inputs from more than 25% of the dorsomedial-to-ventrolateral axis of the medial entorhinal cortex [94],[95]. Therefore, while a single dendritic branch get its *M*≈100 synaptic inputs randomly from nearly 25000 entorhinal cortical neuron, we assume that each synapse on a dendritic branch comes from different entorhinal neurons.

By electrical recordings from different hippocampal regions one can estimate the proportion of simultaneously active cells within a reasonable time window. We call this number the sparseness of the representation in the given area. Specifically, 1–5% of the granule cells are active simultaneously in the DG [24],[29], therefore we used *sp*_DG_ = 0.05. The sparseness of the entorhinal input is somewhat larger, *sp*_EC_ = 0.2 [27],[80],[96].

Experimental data provide a good estimate for the mean firing rate of these neurons, however, they give the variance of the mean across neurons, but not the variance in the firing rate of individual cells. To estimate the variance in the firing rate of an individual cell, we generated random spike trains based on the ISI histogram on Figure 5 of [80]. The expected value and the variance of the number of spikes in a 100 ms time bin (corresponding to one period of the hippocampal theta rhythm) was *μ_EC_*_′_ = 0.32 and 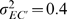 and there was at most 4 spikes during 100 ms in the case of an entorhinal excitatory cell. We scaled these values relative to the maximal firing rate, so we had *μ_EC_* = *μ_EC_*_′_/4 = 0.08 and 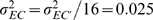 characterizing the distribution of the presynaptic firing *u_j_*. Possible differences in firing statistics across different (medial-lateral or dorsal-ventral) regions in the EC and across individual neurons are neglected here.

Next, we start with originally equal synaptic weights, 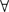 *w_ij_* = *w* = 3 · *μ_EC_*. In this case, if we assume that the firing of entorhinal neurons are independent and identically distributed, we can approximate the total input to a branch with a Gaussian distribution:
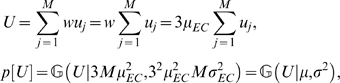
(17)where 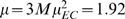 and 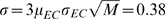. The distribution of the total input *U* is shown on Figure 1C.

### Synaptic Input after Learning

Learning alters the distribution of the total input *U_i_* = Σ*_j_w_ij_u_j_* of dendritic branches (Eq. 17) by modifying synaptic weights. From Eq. 11 used to describe synaptic plasticity, we can see that synaptic weights converge to a fixed point *w_ij_* = *u_j_* whenever the activity of the postsynaptic branch *i* is above threshold *β_d_*. In the stationary state, the weight vector 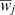 reflects a presynaptic firing pattern 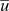. In other words, the learned presynaptic firing pattern is stored in the corresponding synaptic weights.

In order to stimulate initial plasticity in naive branches and prevent learning in those branches that have already learned a pattern, we initialized the synaptic weights to *w_ij_* = *w* = 3*μ_EC_*, which is higher than their expected value at the fixed point (*μ*_EC_). This initialization ensured that the response (*U*) to unlearned inputs decrease during the process of learning, and prevented interference in branches that already have learned a specific pattern. Indeed, synaptic plasticity is enhanced in newly generated granule cells of the hippocampus compared with mature neurons already integrated into functional circuits [36],[64],[97].

After learning we can approximate the distribution of the total synaptic input *U* to a branch by the sum of two Gaussians representing the total input in the case of learned (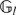) and not learned patterns (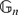), respectively:
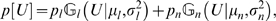
(18)where *p_l_* (*p_n_*) is the probability that one of the branches receive a learned (not learned) input, and *μ_l_* and 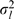 (*μ_n_* and 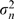) are the mean and the variance of the response to learned (not learned) inputs. If we have a finite number (*N* *_S_*) of different inputs, and each branch learns one of them, then
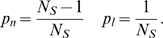
(19)Distribution of the total input to the dendritic branches before and after learning is shown on Figure 3A. Parameters *μ_n_* = 1, *σ_n_* = 0.39, *μ_l_* = 5.6 and *σ_l_* = 0.58 were estimated numerically based on the reconstructed firing characteristics of entorhinal neurons. We assumed that each branches learned one of the samples and the probability that one of the branches receive its learned input (*N/N* *_S_*) was the sparseness in the DG (*sp*_DG_≈0.05. Note, that the distribution of 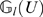 is the theoretical distribution of the responses to learned inputs, from which each branch draw only a few (perhaps one) sample because learning is very sparse.

We recalculated the two functions *H*(*U*) and *K*(*U* ^*^) with the new input distributions by replacing *μ* and *σ* with *μ_n_* and *σ_n_* in Equations 9–10 and 27–28, and by changing the distribution of *U* in Eq. 29 from Eq. 17 to Eq. 18. In these calculations, we neglected the possibility that two (or more) branches may both get their learned input at the same time. Finally, we determined the firing threshold by solving the following integral to *β* (see Eq. 24–25):
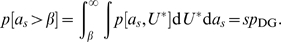
(20)


### Criteria for Independent Firing

In the case of continuous variables we can write that *H*(*U_i_*) = *H*(*U*). The function *H*(*U*) has the form:
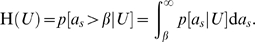
(21)The conditional probability 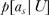 has a form similar to Eq. 8, except that we have only *N*−1 random variables from the Gaussian distribution of *U* (Eq. 17) with parameters *μ_F_* and 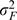, therefore we can write that:
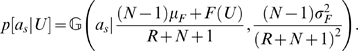
(22)We can compute the second function *K*(*U* ^*^) as follows:
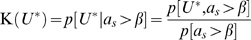
(23)

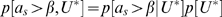
(24)

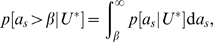
(25)where 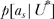 is the conditional distribution of the somatic activation *a_s_* and the maximal dendritic input *U* ^*^. The distribution 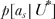 is similar to the distribution of 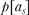 in Eq. 8 with two important differences: First, we have only *N*−1 random variables. Second, we know that 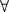 *U*<*U* ^*^, therefore the distribution of the inputs to other branches is different from the Gaussian in Eq. 17. Hence we can write, that
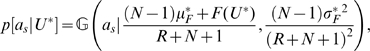
(26)where 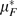 and 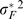 are the conditional expectation and variance of the distribution *p*[*F*(*U*)|*U*<*U* ^*^]. We calculate 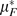 and 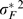 by integrating Equations 9–10 from −∞ to *U* ^*^:
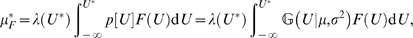
(27)

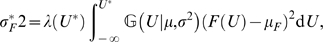
(28)where 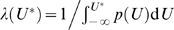 is a normalization factor. Finally we calculate the last term of Eq. 24, the distribution of *U* ^*^ as follows:
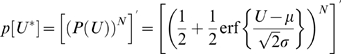
(29)where *P*(*U*) is the cumulative distribution function (CDF) of *U* and [*X*]′ marks derivation. The intuition behind Equation 29 is that: First, *P*(*U*) is the probability that a given input is smaller than *U*. Second, *P*(*U*)*^N^* is the probability that all inputs are smaller than *U*, also the (CDF) of *U* ^*^. Third, its derivative [*P*(*U*)*^N^*]′ gives us the probability density function (PDF) of *U* ^*^. The PDF of *U* is a Gaussian function, its CDF can be expressed with the Gauss error function (erf{}).

### Coupling Between Dendritic Subunits

To calculate the dependence of the dendritic activation on the inputs, we first repeat Eq. 6:
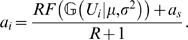
(30)Next, we substitute 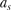 in Equation 30 with Eq. 7:
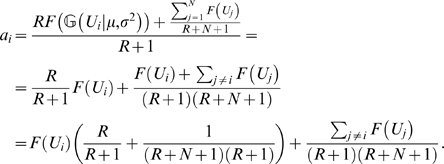
(31)The two terms of the sum in Eq. 31 are independent, because *U_i_* is independent from *U_j_*s, therefore we can calculate the distribution of 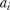 by the convolution of two distributions (corresponding to the two terms in the sum). The second term in Eq. 31 is the sum of independent random variables and we approximate it with a Gaussian (similarly as we did it for 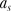 previously, Eq. 8). The distribution of *U_i_* is a Gaussian (Eq. 17), that we can transform into the first term of Eq. 31 by a Jacobian factor [98]:
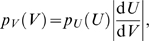
(32)where *V* = *F*(*U*). We get the distribution 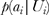 by substituting the first term of Eq. 31 by a Dirac delta distribution. Similarly, we can calculate 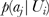 by first computing a conditional sum in the second term (*U_j_*+Σ*_k_*_≠{*i*,*j*}_ *U_k_*) as described by Eq. 22 and then performing the convolution.

The R software environment [99] was used to analyze the data and to prepare the figures.

## Supporting Information

Text S1 The nonlinearity of dendritic integration in a conductance-based model of dentate granule cells.
(0.22 MB PDF)

Text S2 Numerical analysis with sigmoid dendritic integration function.
(0.46 MB PDF)

Text S3 Pattern separation in the dentate gyrus is more effective with multiple fields
(0.26 MB PDF)

Text S4 Calculation of the Integrals of the Different Dendritic Integration Functions
(0.08 MB PDF)
