# Supplementary material for: Parallel Computational Subunits in Dentate Granule Cells Generate Multiple Place Fields
Source: PLoS Comput Biol. 2009 Sep 11;5(9):e1000500. doi: 10.1371/journal.pcbi.1000500 (PMC2730574; doi:10.1371/journal.pcbi.1000500)
Supplement: Text S1 — The nonlinearity of dendritic integration in a conductance-based model of dentate granule cells. (0.22 MB PDF) [file pcbi.1000500.s001.pdf]

Parallel Computational Subunits in Dentate Granule Cells Generate Multiple Place Fields

Balázs Ujfalussy[1,*], Tamás Kiss[1], Péter Érdi[1,2]

1 Dept. Biophysics, KFKI Research Institute for Particle and Nuclear Physics of the Hungarian Academy of Sciences, Budapest, Hungary

2 Center for Complex Systems Studies, Kalamazoo College, Kalamazoo, MI, USA

∗ **E-mail:ubi@rmki.kfki.hu**

Text S1

Conductance-based model

In order to gain more insight into the local dendritic intagration of synaptic inputs and to estimate the dendritic intagration function in hippocampal granule cells we adapted the multicompartmental conductance-based model of Aradi and Holmes (1999) and studied the local responses to dendritic current injections with increasing amplitude. For the present simulations we used the *reduced model* of Aradi and Holmes (1999) which consisted of 28 dendritic, 1 somatic and 31 axonal compartments. In their paper the dendritic tree of the granule cell was modelled by two long, parallel branches with identical morphology (length: 450 μm, diameter: 3 μm, 14 compartments each). We modified the diameter of the branches such that one of them represented a single dendritic branch of a granule cell (diameter: 1.1 μm) while the other represented all other branches (diameter: 4.8 μm). This change of the model's morphology did not modify its passive membrane properties (time constant and input resistance) of the neuron measured at the soma. All other morphological and biophysical parameters, including the passive membrane parameters, channel kinetics and conductances were taken from the original paper [1]. The simulations were performed using the NEURON (version 7.0) simulation environment [2] and the original source code of the model can be downloaded from the ModelDB `http://senselab.med.yale.edu/ModelDB/` .

We injected brief (2 ms) depolarizing current pulses with increasing amplitude to the thinner branch 375 μm from the soma, and the voltage response in the same branch was recorded 320 μm from the soma. Although three different voltage-gated calcium currents (T, N and L type) were included in the dendritic membrane, the voltage responses were near linear for a large range of current amplitudes (0.05-0.5 nA) due to the effect of dendritic potassium currents. However, the elevated level of acetylcholine during

hippocampal theta rhythm [3, 4] has been shown to increase neuronal excitability in the hippocampus by the suppression of potassium channel conductances [5]. Indeed, if the maximal conductance of the dendritic potassium channels were reduced by 10–30% of its original value, than dendritic action potentials were elicited by suprathreshold stimulation (Figure S.1A) and these action potentials propagated actively towards the soma. In this case, the dendritic integration measured by either the maximum of the local voltage response (Figure S.1B) or the electric charge flowing from the excited branch to the soma (Figure S.1C) became highly nonlinear. The dendritic response to longer (20 ms) but weaker (5-50 pA) current pulses was very similar (Figure S.1D-F). Dendritic responses obtained with potassium conductance 30% reduced (red symbols on Figure S.1C) were compared with the dendritic integration functions used in the main article (Figure 1C).

References

1. Aradi I, Holmes WR (1999) Role of multiple calcium and calcium-dependent conductances in regulation of hippocampal dentate granule cell excitability. J Comput Neurosci 6: 215-35.

2. Carnevale T, Hines M (2007) The NEURON book. Cambridge University Press.

3. Kametani H, Kawamura H (1990) Alterations in acetylcholine release in the rat hippocampus during sleep-wakefulness detected by intracerebral dialysis. Life Sci 47: 421-6.

4. Marrosu F, Portas C, Mascia MS, Casu MA, Fa M, et al. (1995) Microdialysis measurement of cortical and hippocampal acetylcholine release during sleep-wake cycle in freely moving cats. Brain Res 671: 329-32.

5. Hasselmo ME (1995) Neuromodulation and cortical function: modeling the physiological basis of behavior. Behav Brain Res 67: 1-27.

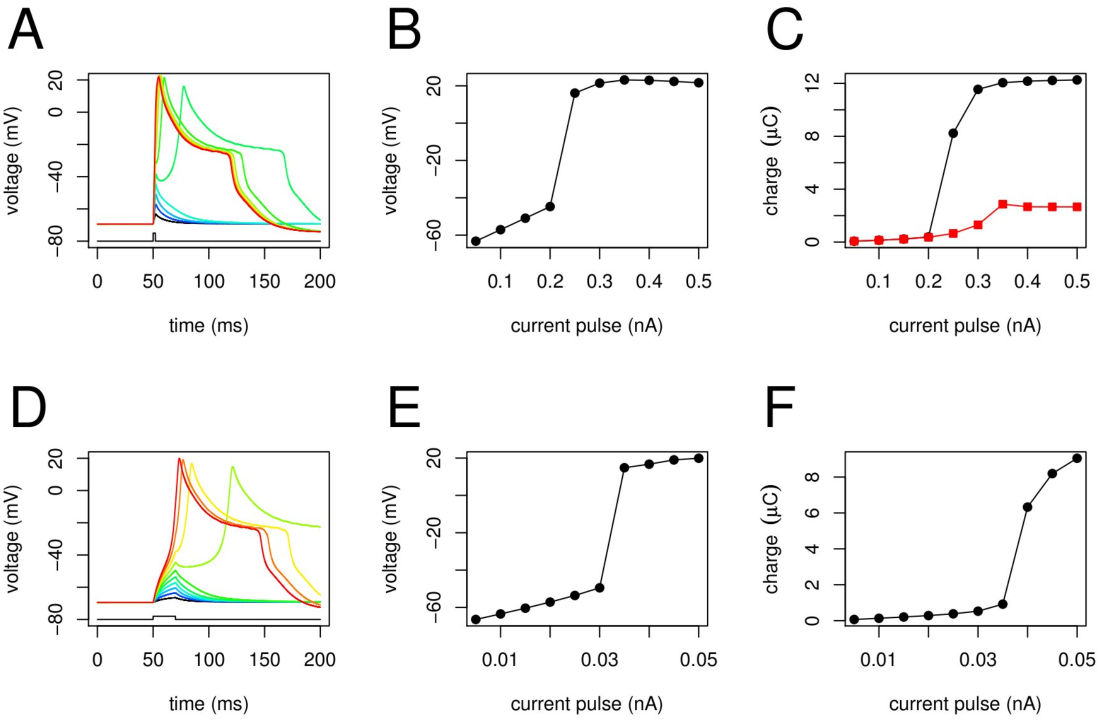

Figure S.1. Dendritic integration in a conductance-based multicompartmental model of hippocampal granule cells. (A) Local voltage responses to brief (2 ms) dendritic current injections with increasing amplitudes (from 0.05 nA, blue, to 0.5 nA, red). Dendritic potassium channels are blocked in all experiments shown in this figure, leaving only 10 % (30 % on the red curve of C) of the original potassium conductance in the dendrite. (B) The maximum of the voltage response is plotted against the amplitude of the injected current. (C) The net charge flowing into the soma from the excited dendrite in the next 50 ms after the current injection. Red squares: the net charge flowing into the soma when K^+-channels are only blocked to allow 30 % of the original potassium conductance (the same data as on Figure 1C). (D-F): The same as on panels (A-C), but with longer (20 ms) and weaker (5-50 pA) current injections.
