# Supplementary material for: Parallel Computational Subunits in Dentate Granule Cells Generate Multiple Place Fields
Source: PLoS Comput Biol. 2009 Sep 11;5(9):e1000500. doi: 10.1371/journal.pcbi.1000500 (PMC2730574; doi:10.1371/journal.pcbi.1000500)
Supplement: Text S2 — Numerical analysis with sigmoid dendritic integration function. (0.46 MB PDF) [file pcbi.1000500.s002.pdf]

Parallel Computational Subunits in Dentate Granule Cells Generate Multiple Place Fields

Balázs Ujfalussy[1,*], Tamás Kiss[1], Péter Érdi[1,2]

1 Dept. Biophysics, KFKI Research Institute for Particle and Nuclear Physics of the Hungarian Academy of Sciences, Budapest, Hungary

2 Center for Complex Systems Studies, Kalamazoo College, Kalamazoo, MI, USA

$*$ **E-mail:ubi@rmki.kfki.hu**

Text S2

Sigmoid integration function

In the present study we approximated the dendritic nonlinearity with a quadratic integration function $F(U) = 0.13U^2$. However, the voltage response in real dendritic trees is bounded, therefore a sigmoid integration function would be a better approximation. Although Equations 9-10 with a sigmoid integration function are analytically intractable, it is easy to sample numerically the input distributions and calculate the resulting dendritic and somatic activations according to Equations 6-7. In order to verify the approximations used during the analytical calculations we first compared the numerical and the analytical results in the case of the quadratic integration function (Figure S.2A-B, 300 000 independent input units innervating 100 neurons, 600 i.i.d. input samples). The difference of input distribution from the ideal Gaussian caused an observable difference between the analytically calculated and the numerically estimated distribution of the maximal dendritic inputs U^*, however, this difference was small, and did not influence our results. Next we chose a sigmoid dendritic integration function $F_S(U) = \frac{3.4}{1 + \exp\left(\frac{4.5-U}{0.3}\right)} + U/4.7$ (Figure S.2C), similar to the input response curve of active dendrites [1–3] (Figure S.1C). The inflexion point of the sigmoid was shifted towards high input values (compare with the input distribution shown in the background on Figure S.2C) indicating that dendritic spikes are rare events caused by extremely strong inputs. Finally we approximated numerically the joint distribution of the somatic activation and the dendritic inputs (Figure S.2D). Learned patterns caused large somatic depolarization therefore the detection probability of dendritic spikes with the sigmoid dendritic integration function was as high as it was in the quadratic case.

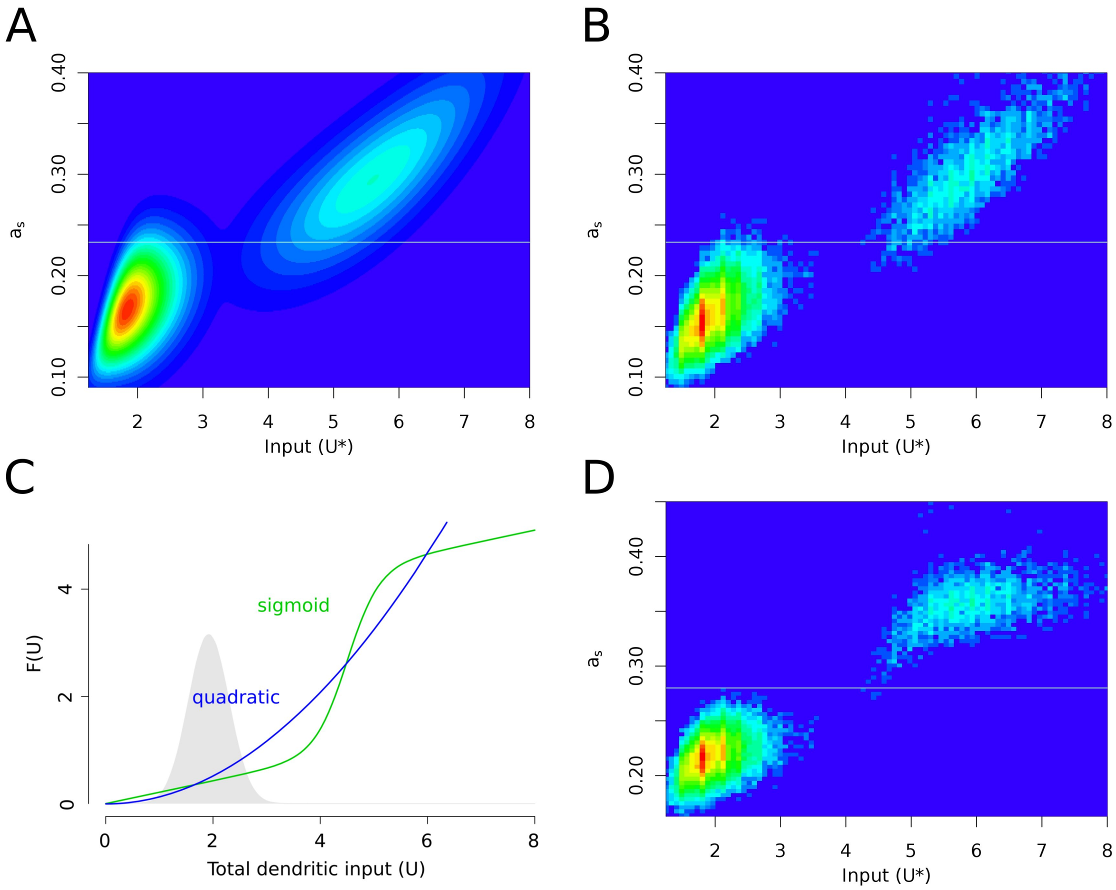

Figure S.2. Numerical analysis with sigmoid dendritic integration function. (A) Analytical results for the joint distribution of the somatic activation a_s, and the maximal dendritic input U^* with the quadratic integration function (replotted from Figure 3D for comparison). (B) The same as on panel A, but with a numerical sample from the input distribution. (C) The sigmoid integration and the quadratic integration functions. The theoretical distribution of the synaptic inputs before learning is shown in the background (replotted from Figure 1C for comparison). (D) The joint distribution of the somatic activation and the maximal dendritic input with the sigmoid dendritic integration function. $N = 30$, $R = 0.01$

References

1. Schiller J, Major G, Koester HJ, Schiller Y (2000) NMDA spikes in basal dendrites of cortical pyramidal neurons. Nature 404: 285-9.

2. Wei DS, Mei YA, Bagal A, Kao JP, Thompson SM, et al. (2001) Compartmentalized and binary behavior of terminal dendrites in hippocampal pyramidal neurons. Science 293: 2272-5.

3. Ariav G, Polsky A, Schiller J (2003) Submillisecond precision of the input-output transformation function mediated by fast sodium dendritic spikes in basal dendrites of CA1 pyramidal neurons. J Neurosci 23: 7750-8.
