# Supplementary material for: Parallel Computational Subunits in Dentate Granule Cells Generate Multiple Place Fields
Source: PLoS Comput Biol. 2009 Sep 11;5(9):e1000500. doi: 10.1371/journal.pcbi.1000500 (PMC2730574; doi:10.1371/journal.pcbi.1000500)
Supplement: Text S3 — Pattern separation in the dentate gyrus is more effective with multiple fields (0.26 MB PDF) [file pcbi.1000500.s003.pdf]

Parallel Computational Subunits in Dentate Granule Cells Generate Multiple Place Fields

Balázs Ujfalussy[1,*], Tamás Kiss[1], Péter Érdi[1,2]

1 Dept. Biophysics, KFKI Research Institute for Particle and Nuclear Physics of the Hungarian Academy of Sciences, Budapest, Hungary

2 Center for Complex Systems Studies, Kalamazoo College, Kalamazoo, MI, USA

∗ **E-mail:ubi@rmki.kfki.hu**

Text S3

Pattern separation with multiple fields

The efficacy of two different model of spatial representation are compared in this section. In the first case, each of the 333 neuron has a single, but relatively large place field randomly located within the environment (Figure S.3, red). In the second model each neuron ($n = 333$) had three, randomly distributed, smaller place fields (Figure S.3, blue). The sparseness of the representation was similar in both cases ($sp_{\mathrm{DG}} = 0.03$). Due to the smaller field sizes, the similarity of the representation of different locations (quantified by the Population Vector correlation [1]) decreased steeper in the second model (Figure S.3) leading to more efficient separation of similar input patterns (nearby locations).

References

1. Leutgeb J, Leutgeb S, Moser M, Moser E (2007) Pattern separation in the dentate gyrus and CA3 of the hippocampus. Science 315: 961-6.

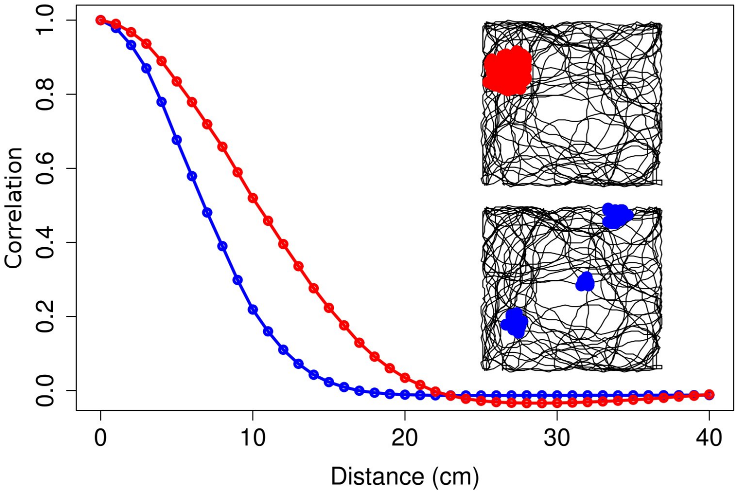

Figure S.3. Pattern separation with multiple fields. Pattern separation is more efficient if neurons have multiple, smaller and randomly located place fields (blue) than if they have a single larger field (red). Inset shows typical examples for the two different cases. The similarity between the neural representation of different locations was quantified by the average correlation between the corresponding cell activities (population vector correlation [1]). The PV correlation decreases faster if neurons have smaller fields, leading to more efficient pattern separation. For example, at two different locations separated by 10 cm the correlation between the neural activity is ≈0.5 with single, larger fields, and ≈0.2 with multiple, smaller fields. The number of neurons ($n = 333$) and the sparsity of the representation ($sp_{\mathrm{DG}} = 0.03$) was similar in both cases.
