# Supplementary material for: Parallel Computational Subunits in Dentate Granule Cells Generate Multiple Place Fields
Source: PLoS Comput Biol. 2009 Sep 11;5(9):e1000500. doi: 10.1371/journal.pcbi.1000500 (PMC2730574; doi:10.1371/journal.pcbi.1000500)
Supplement: Text S4 — Calculation of the Integrals of the Different Dendritic Integration Functions (0.08 MB PDF) [file pcbi.1000500.s004.pdf]

Parallel Computational Subunits in Dentate Granule Cells Generate Multiple Place Fields

Balázs Ujfalussy[1,*], Tamás Kiss[1], Péter Érdi[1,2]

1 Dept. Biophysics, KFKI Research Institute for Particle and Nuclear Physics of the Hungarian Academy of Sciences, Budapest, Hungary

2 Center for Complex Systems Studies, Kalamazoo College, Kalamazoo, MI, USA

∗ E-mail:ubi@rmki.kfki.hu

Text S4

Calculation of the Integrals of the Different Integration Functions

In this section we compute the integrals in Equations 10-11 for the two different dendritic integration functions shown on Figure 1. To make the comparisons easier, we scaled both functions to have the same expected value ($\mathrm{E}[F(U)] = 0.5$).

S.1 Linear Integration Function

In the linear case, we use the $F_L(U) = 0.26U$ integration function. First we calculate the expected value of the function, if the distribution of U is from Eqn. 18: $p[U] = \mathbb{G}(U|\mu, \sigma^2)$.

$$
\begin{aligned}
\mu_L &= \textstyle\int_{-\infty}^{\infty} F_L(U)\mathbb{G}(U|\mu,\sigma^2)\mathrm{d}U = \\
&= \int_{-\infty}^{\infty} \frac{0.26U}{\sqrt{2\pi\sigma^2}} \exp\left\{-\frac{(U-\mu)^2}{2\sigma^2}\right\} \mathrm{d}U = \qquad\qquad z = \tfrac{U-\mu}{\sqrt{2}\sigma} \\
&= \frac{0.26}{\sqrt{\pi}} \int_{-\infty}^{\infty} (z\sqrt{2}\sigma + \mu) \exp\left\{-z^2\right\} \mathrm{d}z = \\
&= \frac{0.26\sqrt{2}\sigma}{\sqrt{\pi}} \int_{-\infty}^{\infty} z \exp\left\{-z^2\right\} \mathrm{d}z + \frac{0.26\mu}{\sqrt{\pi}} \int_{-\infty}^{\infty} \exp\left\{-z^2\right\} \mathrm{d}z.
\end{aligned}
\tag{S.1}
$$

By changing the upper limit of the integrand from ∞ to U^* (z^*), we get the conditional expectations (Eqn. 27). We used the integration rules in Eqn. S.11 and S.12 to compute the primitive function of Eqn. S.1:

$$\mu_L^* = \frac{0.26\Lambda(z^*)}{\sqrt{\pi}} \left(\sqrt{2}\sigma \left[-\frac{1}{2}\exp\{-z^{*2}\} + \frac{1}{2}\exp\{-(-\infty)^2\} \right] + \mu \left[\frac{\sqrt{\pi}}{2}\mathrm{erf}\{z^*\} - \frac{\sqrt{\pi}}{2}\mathrm{erf}\{-\infty\} \right] \right), \tag{S.2}$$

where $\Lambda(z^*) = 1/\int_{-\infty}^{z^*} p(z)\mathrm{d}z$ is a normalization factor. Of course, we can use that $\exp\{-\infty\} = 0$, $\mathrm{erf}\{-\infty\} = -1$ and $\mathrm{erf}\{\infty\} = 1$. By substituting z^* with ∞, as in Eqn. S.1, we have:

$$\mu_L = \frac{0.26}{\sqrt{\pi}} \left(\sqrt{2}\sigma\,[0] + \mu \left[\frac{\sqrt{\pi}}{2} + \frac{\sqrt{\pi}}{2} \right] \right) = 0.26\mu. \tag{S.3}$$

We can calculate the variance in a similar way:

$$\sigma_L^2 = \int_{-\infty}^{\infty} \mathbb{G}(U|\mu,\sigma^2)(F_L(U) - \mu_L)^2 \mathrm{d}U =$$

$$= \int_{-\infty}^{\infty} \frac{(0.26U - \mu_L)^2}{\sqrt{2\pi\sigma^2}} \exp\left\{ -\frac{(U-\mu)^2}{2\sigma^2} \right\} \mathrm{d}U = \qquad U = \sqrt{2}\sigma z + \mu$$

$$= \frac{1}{\sqrt{\pi}} \int_{-\infty}^{\infty} (0.26\sqrt{2}\sigma z + 0.26\mu - \mu_L)^2 \exp\left\{-z^2\right\} \mathrm{d}z = \qquad 0.26\mu - \mu_L = \Delta\mu$$

$$= \frac{1}{\sqrt{\pi}} \int_{-\infty}^{\infty} (0.26^2 2\sigma^2 z^2 + 0.52\sqrt{2}\sigma z\Delta\mu + \Delta\mu^2) \exp\left\{-z^2\right\} \mathrm{d}z =$$

$$= \frac{0.26^2 2\sigma^2}{\sqrt{\pi}} \int_{-\infty}^{\infty} z^2 \exp\left\{-z^2\right\} \mathrm{d}z + \frac{0.52\sqrt{2}\sigma\Delta\mu}{\sqrt{\pi}} \int_{-\infty}^{\infty} z \exp\left\{-z^2\right\} \mathrm{d}z + \frac{\Delta\mu^2}{\sqrt{\pi}} \int_{-\infty}^{\infty} \exp\left\{-z^2\right\} \mathrm{d}z. \tag{S.4}$$

And the primitive function of Equation S.4 again to z^*, by using Eqn. S.11,S.12,S.13:

$$\sigma_L^{*\,2} = \Lambda(z^*) \left[\frac{0.26^2\sigma^2}{\sqrt{\pi}} \left(\frac{\sqrt{\pi}}{2}\mathrm{erf}\{z^*\} - z^*\exp\{-z^{*2}\} + \frac{\sqrt{\pi}}{2} \right) \right] - \Lambda(z^*) \left[\frac{0.26\sqrt{2}\sigma\Delta\mu^*}{\sqrt{\pi}} \exp\{-z^{*2}\} + \frac{\Delta\mu^{*2}}{2} \left(\mathrm{erf}\{z^*\} + 1\right) \right], \tag{S.5}$$

where we used that $\lim_{x\to\infty} \exp\{-x^2\} = 0$, $\lim_{x\to-\infty} \mathrm{erf}\{x\} = -1$, $\lim_{x\to\infty} \mathrm{erf}\{x\} = 1$ and $\lim_{x\to\infty} x\exp\{-x^2\} = 0$. Here $\Delta\mu^* = 0.26\mu - \mu_L^*$. Substituting z^* by ∞, we got that:

$$\sigma_L^2 = 0.26^2\sigma^2 - 0 + \Delta\mu^{*2} = 0.26^2\sigma^2. \tag{S.6}$$

S.2 Quadratic Integration Function

In the quadratic case, we chose the $F_Q(U) = kU^2$ integration function, with $k = 0.13$, where $F_Q(0) = 0$ and $\mu_Q \approx 0.5$, as we show below.

$$\mu_Q = \int_{-\infty}^{\infty} F_Q(U)\mathbb{G}(U|\mu,\sigma^2)\mathrm{d}U = \int_{-\infty}^{\infty} \frac{kU^2}{\sqrt{2\pi\sigma^2}} \exp\left\{-\frac{(U-\mu)^2}{2\sigma^2}\right\}\mathrm{d}U = \qquad z = \tfrac{U-\mu}{\sqrt{2}\sigma}$$
$$= \frac{k}{\sqrt{\pi}} \int_{-\infty}^{\infty} \left(\sigma\sqrt{2}z + \mu\right)^2 \exp\{-z^2\}\mathrm{d}z =$$
$$= \frac{2k\sigma^2}{\sqrt{\pi}} \int_{-\infty}^{\infty} z^2 \exp\{-z^2\}\mathrm{d}z + \frac{2k\sqrt{2}\sigma\mu}{\sqrt{\pi}} \int_{-\infty}^{\infty} z\exp\{-z^2\}\mathrm{d}z + \frac{k\mu^2}{\sqrt{\pi}} \int_{-\infty}^{\infty} \exp\{-z^2\}\mathrm{d}z. \tag{S.7}$$

The integration of Eqn S.7 using Eqns. (S.11,S.12-S.13) leads to:

$$\mu_Q^* = k\Lambda(z^*)\left[\frac{\sigma^2}{\sqrt{\pi}}\left(\frac{\sqrt{\pi}}{2}\mathrm{erf}\{z^*\} - z^*\exp\{-z^{*2}\} + \frac{\sqrt{\pi}}{2}\right) - \frac{\sqrt{2}\sigma\mu}{\sqrt{\pi}}\exp\{-x^{*2}\} + \frac{\mu^2}{2}(\mathrm{erf}\{z^*\}+1)\right]. \tag{S.8}$$

Substituting z^* with ∞ in Equation S.8, we got that $\mu_Q = k(\mu^2 + \sigma^2)$. We chose the parameter $k = 0.13$, then $\mu_Q = 0.498 \approx 0.5$. And the variance:

$$\sigma_Q^2 = \int_{-\infty}^{\infty} \frac{(kU^2 - \mu_Q)^2}{\sqrt{2\pi\sigma^2}} \exp\left\{-\frac{(U-\mu)^2}{2\sigma^2}\right\}\mathrm{d}U = \qquad z = \tfrac{U-\mu}{\sqrt{2}\sigma}$$
$$= \frac{1}{\sqrt{\pi}} \int_{-\infty}^{\infty} (k(\sigma\sqrt{2}z+\mu)^2 - \mu_Q)^2 \exp\left\{-z^2\right\}\mathrm{d}z$$

$$c = k\sigma^2 \tag{S.9}$$
$$= \frac{1}{\sqrt{\pi}} \int_{-\infty}^{\infty} (2k\sigma^2 z^2 + 2k\sqrt{2}\sigma\mu z + k\mu^2 - \mu_Q)^2 \exp\left\{-z^2\right\}\mathrm{d}z = \qquad d = 2k\sqrt{2}\sigma\mu$$
$$k\mu^2 - \mu_Q = e$$
$$= \frac{1}{\sqrt{\pi}} \int_{-\infty}^{\infty} (4c^2z^4 + 4cdz^3 + (d^2+4ce)z^2 + 2dez + e^2)\exp\left\{-z^2\right\}\mathrm{d}z =$$

The integration of Eqn S.9 using Eqns. (S.11,S.12-S.15) leads to:

$$
\begin{aligned}
{\sigma_Q^*}^2 = \Lambda(z^*) \Bigg[\frac{c^2}{\sqrt{\pi}} \left(\left(-2{z^*}^3 - 3z^* \right) \exp\{-{z^*}^2\} + \frac{3\sqrt{\pi}}{2}\mathrm{erf}\{z^*\} + \frac{3\sqrt{\pi}}{2} \right) \\
- \frac{2cd}{\sqrt{\pi}} \exp\{-{z^*}^2\}({z^*}^2 + 1) \\
+ \frac{d^2 + 4ce}{\sqrt{\pi}} \left(\frac{\sqrt{\pi}}{4}\mathrm{erf}\{z^*\} - \frac{z^*}{2} \exp\{-{z^*}^2\} + \frac{\sqrt{\pi}}{4} \right) \\
- \frac{de}{\sqrt{\pi}} \exp\{-{z^*}^2\} + \frac{e^2}{2}(\mathrm{erf}\{z^*\} + 1) \Bigg] .
\end{aligned}
\tag{S.10}
$$

Substituting z^* with ∞ in Equation S.10, we got that $\sigma_Q^2 = 2c^2 + d/2 = 0.0348$.

Integrals

To compute the above integrals, we used the following integration rules:

$$\int \exp\left\{-x^2\right\} \mathrm{d}x = \frac{\sqrt{\pi}}{2}\mathrm{erf}\{x\} \tag{S.11}$$

$$\int x \exp\left\{-x^2\right\} \mathrm{d}x = -\frac{1}{2} \exp\{-x^2\} \tag{S.12}$$

$$\int x^2 \exp\left\{-x^2\right\} \mathrm{d}x = \frac{\sqrt{\pi}}{4}\mathrm{erf}\{x\} - \frac{1}{2} x \exp\{-x^2\} \tag{S.13}$$

$$\int x^3 \exp\left\{-x^2\right\} \mathrm{d}x = -\frac{1}{2} \exp\{-x^2\}(x^2 + 1) \tag{S.14}$$

$$\int x^4 \exp\left\{-x^2\right\} \mathrm{d}x = \left(-\frac{x^3}{2} - \frac{3x}{4} \right) \exp\{-x^2\} + \frac{3\sqrt{\pi}}{8}\mathrm{erf}\{x\} \tag{S.15}$$
